# Chronic Hepatitis C Pathogenesis: Immune Response in the Liver Microenvironment and Peripheral Compartment

**DOI:** 10.3389/fcimb.2021.712105

**Published:** 2021-08-03

**Authors:** Daniela Alejandra Rios, Paola Cecilia Casciato, María Soledad Caldirola, María Isabel Gaillard, Cecilia Giadans, Beatriz Ameigeiras, Elena Noemí De Matteo, María Victoria Preciado, Pamela Valva

**Affiliations:** ^1^Laboratory of Molecular Biology, Multidisciplinary Institute for Investigation in Pediatric Pathologies (IMIPP), CONICET-GCBA, Pathology Division, Ricardo Gutiérrez Children’s Hospital, Buenos Aires, Argentina; ^2^Liver Unit, Italian’s Hospital of Buenos Aires, Buenos Aires, Argentina; ^3^Immunology Unit, Multidisciplinary Institute for Investigation in Pediatric Pathologies (IMIPP), CONICET-GCBA, Ricardo Gutiérrez Children’s Hospital, Buenos Aires, Argentina; ^4^Liver Unit, Ramos Mejía Hospital, Buenos Aires, Argentina

**Keywords:** immunopathogenesis, HCV: Hepatitis C virus, chronic liver disease, inflammatory liver infiltrate, Treg: regulatory T lymphocytes, Th17, peripheral blood

## Abstract

Chronic hepatitis C (CHC) pathogenic mechanisms as well as the participation of the immune response in the generation of liver damage are still a topic of interest. Here, we evaluated immune cell populations and cytokines in the liver and peripheral blood (PB) to elucidate their role in CHC pathogenesis. B, CTL, Th, Treg, Th1, Th17, and NK cell localization and frequency were evaluated on liver biopsies by immunohistochemistry, while frequency, differentiation, and functional status on PB were evaluated by flow cytometry. TNF-α, IL-23, IFN-*γ*, IL-1β, IL-6, IL-8, IL-17A, IL-21, IL-10, and TGF-β expression levels were quantified in fresh liver biopsy by RT-qPCR and in plasma by CBA/ELISA. Liver CTL and Th1 at the lobular area inversely correlated with viral load (r = −0.469, *p* =0.003 and r = −0.384, *p* = 0.040). Treg correlated with CTL and Th1 at the lobular area (r = 0.784, *p* < 0.0001; r = 0.436, *p* = 0.013). Th17 correlated with hepatic IL-8 (r = 0.52, *p* < 0.05), and both were higher in advanced fibrosis cases (Th17 *p* = 0.0312, IL-8 *p* = 0.009). Hepatic cytokines were higher in severe hepatitis cases (IL-1β *p* = 0.026, IL-23 *p* = 0.031, IL-8 *p* = 0.002, TGF-β, *p*= 0.037). Peripheral NK (*p* = 0.008) and NK *dim* (*p* = 0.018) were diminished, while NK *bright* (*p* = 0.025) was elevated in patients *vs.* donors. Naïve Th (*p* = 0.011) and CTL (*p* = 0.0007) were decreased, while activated Th (*p* = 0.0007) and CTL (*p* = 0.0003) were increased. IFN-*γ* production and degranulation activity in NK and CTL were normal. Peripheral cytokines showed an altered profile *vs.* donors, particularly elevated IL-6 (*p* = 0.008) and TGF-β (*p* = 0.041). Total hepatic CTLs favored damage. Treg could not prevent fibrogenesis triggered by Th17 and IL-8. Peripheral T-lymphocyte differentiation stage shift, elevated cytokine levels and NK-cell count decrease would contribute to global disease.

## Introduction

Chronic hepatitis C (CHC) is a progressive disease that may result in cirrhosis and/or hepatocellular carcinoma; therefore liver failure because of hepatitis C virus (HCV) infection is one of the most common reasons for liver transplantation (Manns et al., 2017).

CHC still represents a major global health problem affecting approximately 71 million people worldwide (Polaris Observatory HCV Collaborators, 2017); however, the real burden of HCV infection displays great uncertainty since most infected people remain undiagnosed and untreated. In this context one of the major current challenges is to carry out screening programs to assess CHC in the context of an asymptomatic infection (Hollande et al., 2020). Accordingly, the WHO goal of eliminating HCV by 2030 is based on three main actions: strengthening and increasing outreach screening; increasing access to treatment; and improving prevention (Hollande et al., 2020).

Given that there is no protective vaccine approved, the highly effective direct-acting antiviral agents (DAAs) are of great importance since they reach a dramatically high-sustained virological response (SVR) rate. Nevertheless, they cannot fully eradicate hepatocellular carcinoma risk, especially in HCV-cured patients with advanced liver disease, suggesting an accumulation of irreversible damages to the liver during long-term HCV infection (Reig et al., 2017; Goto et al., 2020; Massoud, 2020; Sangiovanni et al., 2020). Therefore, knowledge of the CHC pathogenic mechanisms as well as of the participation of the immune response in the generation of liver damage (Tang et al., 2016; Manns et al., 2017) is still a topic of interest.

It is well known that HCV poses a constant challenge to liver homeostasis, causing stress and inflammation (Goto et al., 2020). The liver microenvironment is extremely complex with numerous immune cell populations that, along with the cytokines they produce, play a central role in viral elimination; thus, the interplay between virus and host immune response may influence infection outcome (Mengshol et al., 2007; Valva et al., 2014; Rios et al., 2017). Remarkably, in the chronic stage the role of the immune cells becomes more complex since their functionality alteration would contribute to liver damage (Jenne and Kubes, 2013). However, the results in this regard are controversial, and the underlying mechanism by which various populations would be involved is still under discussion.

Natural killer (NK) cells, major cellular components of the antiviral innate immune system, display an important role in CHC pathogenesis. NK cells could be classified into two different subsets: NK *dim* cells are more cytolytic in nature, whereas NK *bright* cells usually have a predominant cytokine-producing phenotype (Cooper et al., 2001). These cells would exert a dual role in the pathogenesis: they could increase inflammation as a consequence of the elimination of infected hepatocytes, and they would also be involved in the regulation of the fibrogenic process (Mengshol et al., 2007; Fasbender et al., 2016; Njiomegnie et al., 2020).

T cells display a crucial role in determining spontaneous resolution *versus* virus persistence during acute infection. On one hand, cytotoxic T lymphocytes (CTL, CD8+ T cells) are essential effectors for the control of HCV infection since they participate in the elimination of infected cells by releasing cytotoxic granules and the expression of cell death-inducing receptor ligands. They also inhibit viral replication through non-cytolytic mechanisms, such as IFN-**γ** and TNF-*α* secretion. However, it is postulated that in the chronic stage HCV-specific CTLs would be functionally impaired with respect to IFN-**γ** production, proliferation, cytotoxicity, and degranulation potential (Wedemeyer et al., 2002; Neumann-Haefelin and Thimme, 2013; Heim and Thimme, 2014). For its part, T helper lymphocytes (Th, CD4+ T cells) act as central regulators of the adaptive immune response through augmenting CTL response and antigen-specific B lymphocytes, although they also seem to have altered functions and decreased proliferation activity during chronicity. It is well known that Th can be induced to differentiate towards Th1, Th17, or Treg cells, with distinct phenotypes and functions, depending on the stimuli received through their TCR, as well as signals from the cytokine milieu (Schmitt and Ueno, 2015). Moreover, recent *in vitro* studies showed that these differentiation stages are not definitive, but rather flexible in response to changes that occur in the microenvironment.

Th1 lymphocytes, which express the transcription factor Tbet, are considered mainly pro-inflammatory since they participate in cell-mediated immune response by secreting IFN-γ, TNF-α, and IL-2 that stimulate macrophages and CTL function (Schmitt and Ueno, 2015; DuPage and Bluestone, 2016), and their differentiation is promoted by IL-12 and IFN-*γ*. Treg lymphocytes, expressing CD25 and Foxp3, are considered immune response and inflammation suppressors since they act on the maintenance of self-tolerance by direct cell–cell contact mechanisms and through the secretion of anti-inflammatory cytokines such as IL-10 and TGF-*β* (Lu et al., 2017). The peripheral blood Treg counts in CHC patients are variable, (Barjon et al., 2015) but liver Tregs in CHC patients are increased in comparison to those in non-infected individuals or to those who resolved HCV infection (Claassen et al., 2010). Notably, the precise role of Tregs in liver injury control is not clearly defined, since some studies described a high Treg frequency in cases with milder fibrosis (Claassen et al., 2010), while others reported association with more severe fibrosis stages (Langhans et al., 2013). Lastly, despite their opposite functional properties, Th17 cells and Tregs share similar developmental requirements, namely, the pleiotropic cytokine TGF-*β*. The key is the concentration-dependent function of TGF-*β*; at low concentrations TGF-*β* synergizes with IL-6 and IL-21 to promote IL-23 receptor (IL-23R) expression, favoring Th17 differentiation, whereas at high concentrations, TGF-*β* represses IL-23R and favors Foxp3+ Treg cells (Omenetti and Pizarro, 2015). For its part, Th17 subpopulation is characterized by ROR-**γ** expression and IL-17A/F, IL-21, and IL−22 secretion (Schmitt and Ueno, 2015; DuPage and Bluestone, 2016), which are considered mainly pro-inflammatory since they favor the recruitment of other immune inflammatory cells. Th17 lymphocytes have been implicated in the pathogenesis of other liver pathologies of different etiologies such as alcoholic hepatitis, primary biliary cirrhosis, and chronic hepatitis B (Veldhoen, 2017). As the balance between Th17 and Treg cells is very important for the immune homeostasis maintenance, and its dysfunction in liver has been proved to be associated with hepatic injury and disease, the study of Th17/Treg balance in chronic HCV infection is of great interest for understanding the pathogenesis.

On the other hand, investigations of patient samples instead of animal models are yet needed, since it is crucial to explore different aspects of the disease in its actual context. Likewise, the study of liver biopsies is currently infrequent given that tests are usually carried out in peripheral blood, but this does not necessarily reflect what happens at the site of infection. Therefore, in this study, we evaluated cellular and immune markers that may be involved in liver damage, allowing a comprehensive liver microenvironment analysis that will contribute to understanding the possible role of the immune system in CHC pathogenesis. Moreover, the peripheral compartment was also analyzed to get an integrated picture of this pathological condition.

## Materials and Methods

### Ethics Statement

The ethics committees of the hospitals reviewed and approved this study, which is in accordance with the human experimentation guidelines of the institutions and with the Helsinki Declaration of 1975, as revised in 1983. Written informed consent was obtained from all patients.

### Patients and Samples

Liver biopsies and concomitant peripheral blood samples were collected from 48 adult naïve of treatment patients with CHC infection who attended the Hospital Italiano de Buenos Aires and Hospital JM. Ramos Mejía. Liver biopsies were divided into two portions: one fragment was formalin-fixed and paraffin-embedded (FFPE) and the other was conserved in Trizol at −70°C. The presence of anti-HCV antibodies in serum samples and HCV RNA in plasma in at least two separate occasions confirmed CHC infection (2002). Patients documented no other causes of liver disease, autoimmune or metabolic disorders, hepatocellular carcinoma or co-infection with HBV and/or HIV. Alcohol consumption (men >30 g/day; women >20 g/day) was applied as an exclusion criteria.

The clinical and biochemical data from patients (age, gender, risk factor for infection, viral load, genotype, AST and ALT values) were obtained from the medical records.

Peripheral blood samples from 40 healthy donors [age median (min.–max.) 40 years (27–71); gender Male : Female 1:1.05] without any known systemic or liver disease and/or HIV, and with normal biological liver test as well as with absence of anti-HCV antibodies, were also included as uninfected controls for the flow cytometry assays.

**Supplementary Table S1** shows the detailed clinical, virological, and histological features of each HCV adult patient.

### Histological Analysis

Histological sections were blindly evaluated by a pathologist (ENDM). Inflammatory activity and fibrosis were assessed using the modified Knodell scoring system (Histological Activity Index, HAI) and METAVIR (Theise et al., 2012).

### Immunohistochemical Analysis

#### HCV Liver Staining

Infected hepatocytes were evidenced by immunohistochemical detection of viral NS3 protein. Epitope retrieval was performed with sodium citrate buffer (0.01 M, pH 6) in an autoclave for 3 min (20 psi). Endogenous biotin was blocked with Biotin Blocking System (Avidin/Biotin Blocking Kit, Vector Laboratories Inc, Burlingame, CA, USA). The primary mouse antibody for NS3 detection (1:25, clone MMM33, Abcam, Cambridge, UK) was incubated for 1 h at 25°C, and staining was obtained by applying the streptavidin–biotin peroxidase (SBP) system and substrate-chromogen reagent (Vectastain Elite ABC and DAB Substrate Kit for Peroxidase, Vector Laboratories Inc, Burlingame, CA, USA). Immunostained and total hepatocytes were counted in 20 high-power fields (×1,000). No labelling was observed without NS3 primary antibody, with isotype control or on chronic HBV infected liver samples.

#### Characterization and Quantification of the Inflammatory Liver Infiltrate

Infiltrate characterization was performed using appropriate antibodies: mouse anti-CD20 (L26, VENTANA, Roche, Basel, Switzerland), rabbit anti-CD8 (SP57, VENTANA, Roche, Basel, Switzerland), rabbit anti-CD56 (MRQ-42, VENTANA, Roche, Basel, Switzerland), rabbit anti-CD4 (SP35, VENTANA, Roche, Basel, Switzerland), mouse anti-Foxp3 (236A/E7, Abcam, Cambridge, UK), mouse anti-Tbet (4B10, BD Pharmingen, San Jose, CA, USA), and goat anti-IL-17A (AF-317-NA, R&D Systems, Minneapolis, MN, USA). After epitope retrieval [sodium citrate buffer (0.01 M, pH 6) in autoclave during 5 min (20 psi)], sections were incubated with each primary antibody and stained by applying PolyTek HRP anti-Mouse Polymerized Imaging System (PIR080, ScyTek Laboratories, Utah, USA), ultraView™ Universal DAB (cat. 760-500, VENTANA, Roche, Basel, Switzerland) or Cell & Tissue Staining Goat Kit (cat. CTS008, R&D Systems, Minneapolis, MN, USA) as appropriate according to the instructions of the manufacturer. Tonsil sections were used as positive controls, and isotype controls were performed. Immunostained and total cells with lymphocyte morphology were counted in all portal tracts of the tissue section (×400), and frequencies were calculated as positive/total of the whole specimen. Immunostained cells were also counted in 10 random fields from lobular areas, and the results were expressed as immunostained cells/field (×400).

### Quantitative qRT-PCR Analysis

Total RNA was isolated from liver samples using Epicentre Master Pure RNA Purification kit (Illumina, San Diego, CA, USA), according to the instructions of the manufacturer. A DNAse (RQ1 RNAse-free DNAse, Promega, Madison, WI, USA) treatment in all RNA samples was performed. The cDNA was reversed transcribed from 2 μg of RNA, using random 6-mer oligonucleotides (5 ng/μl) and Superscript II RT kit (Invitrogen, Waltham, MA, USA).

The design and validation of TNF-α, IL-23, IFN-*γ*, IL-1β, IL-6, IL-8, IL-17A, IL-21, IL-10 and TGF-*β* specific primers are described in **Supplementary Table S2**. A 1/10 aliquot of the cDNA reaction product (50 ng input) was used in each duplicate qPCR reactions. qPCR was performed in a final volume of 25 µl of Fast Start Universal Sybr Green Master Mix (Roche Diagnostics GmbH, Mannheim, Germany) including 5 µl of diluted cDNA using a StepOne real-time (Applied Biosystems, Foster City, CA, USA). The endogenous HPRT or *β*-actin genes were used as endogenous controls for sample normalization (reference gene) according to the expression level of the studied gene. The normalized transcription values were calculated by the Pfaffl Method (Pfaffl, 2001). Results were expressed as fold change (FC).

### Flow Cytometric Analysis

#### Quantification of Peripheral Lymphocyte Populations

B lymphocytes (CD3−CD19+), CTL (CD3+CD8+), NK (CD3−CD56+), and Th cell (CD3+CD4+) frequencies were assessed using anti-CD45-500 (HI30, Bioscience, San Jose, CA, USA), anti-CD3-APC (SK7, Bioscience, San Jose, CA, USA), anti-CD19-PE-Cy7 (SJ25C1, BD Bioscience, San Jose, CA, USA), anti-CD8-APC-H7 (SK1, BD Bioscience, San Jose, CA, USA), CD56-PE (N901, Beckman Coulter, Chaska, MN, USA), and anti-CD4-V450 (RPA-4, BD Bioscience, San Jose, CA, USA) on fresh heparinized blood samples. Later, NK cells were discriminated in *dim* and *bright* subpopulations according to CD56 expression level.

PBMCs were isolated by Ficoll-Paque (Amersham Bioscience, Buckinghamshire, UK) to assess Th subpopulations. Treg (CD4+/CD25hi/Foxp3+) cells were evaluated using Foxp3 staining kit (cat 560133, BD Pharmingen, San Jose, CA, USA), while Th1 (CD4+/IFN-*γ*+) and Th17 (CD4+/IL-17A+) using Human Th1/Th2/Th17 Phenotyping Kit (cat 560751, BD Pharmingen, San Jose, CA, USA), according to the instructions of the manufacturer. Th1 and Th17 staining was performed on both basal PBMCs and anti-CD3 (0.166 ng/µl); IL-2 (0.08 pg/µl) stimulated PBMCs for 18 h in the presence of Golgi-Stop^®^ (BD Pharmingen, San Jose, CA, USA). Gating strategies are shown in **Supplementary Material**.

Data were collected on a BD FACSCantoTM II cytometer (BD Biosciences, San Jose, CA, USA) and analyzed using BD FACSDiva ™ Software. The analysis was performed with the FlowJo 7.6.2, and the results were expressed as counts in percentage (%) and absolute values (number of cells/µl) and, depending on the test, as nMFI (‘Median Fluorescence Intensity’).

#### T Lymphocyte Differentiation Status Assessment

To define T lymphocytes differentiation status, both CTL and Th lymphocyte subsets were distinguished from CD3+CD8+ and CD3+CD4+ gate, respectively as follows: activated T lymphocytes (HLA-DR+), naïve (N; CD45RA+CD27+), central memory (CM; CD45RA−CD27+), effector memory (EM; CD45RA−CD27−) and effectors (E; CD45RA+CD27−) were evaluated using anti-CD45RA-FITC (L48, BD Bioscience, San Jose, CA, USA), anti-CD3-APC (SK7, BD Bioscience, San Jose, CA, USA), anti-CD8-APCH7 (SK1, BD Bioscience, San Jose, CA, USA), anti-CD4-V450 (RPA-T4, BD Bioscience, San Jose, CA, USA), anti-CD27-PerCP-Cy ™ 5.5 (L128, BD Bioscience, San Jose, CA, USA), and anti-HLA-DR-PE (L243, Beckman Coulter, Chaska, MN, USA). Gating strategies are shown in **Supplementary Material**. For each status (N, CM, EM, E, activated) the results were expressed as counts in percentage (%) in relation to the Th and the CTL.

#### Functional Characterization of CTLs and NK Cells

IFN-*γ* production and degranulation activity were evaluated in both CTL and NK cells.

The IFN-*γ*-producing CTL subset was evaluated by means of a modification of the Th17/Th1 lymphocyte assay; for this purpose, anti-CD8-APC-H7 antibody (SK1, eBioscience, San Diego, CA, USA) was added to the precast antibody cocktail. On the other hand, IFN-*γ* production by NK cells was performed in a separate assay since it requires a different stimulus. Briefly, PBMCs were cultured 16 h in supplemented medium in the absence [basal tube] or presence of rIL−12 (10 ng/ml; eBioscience, San Diego, CA, USA), rIL−15 (2 ng/ml; eBioscience, San Diego, CA, USA), and rIL−18 (10 ng/ml; eBioscience, San Diego, CA, USA) [stimulated tube]. During the last 4 h, Golgi-Stop^®^ (BD Pharmingen, San Jose, CA, USA) was added to the cultures, and then immunostaining was performed using: anti-CD56-APC (N901, Beckman Coulter, Chaska, MN, USA), anti-CD3-PE-Cy7 (UCHT1, BD Pharmingen, San Jose, CA, USA), and anti-IFN-*γ*-PE (4S.B3, BD Pharmingen, San Jose, CA, USA).

The degranulation activity was determined by evaluating CD107a (LAMP-1) expression in PBMCs (Betts et al., 2003; Alter et al., 2004). Briefly, PBMCs were cultured in 1 ml of supplemented medium for 18 h at 37°C 5% CO_2_. Then, cell suspension was separated into four tubes: isotype tube, basal tube, NK tube, and CTL tube. In the NK tube, K562 cells (a human cell line that does not express MHC I molecules) were added as stimulus to evaluate the degranulation activity of NK cells, while in the CTL tube, anti-CD3 (0.33 μg/μl) was added as a stimulus. After 3 h of incubation at 37°C 5% CO_2_, immunostaining using anti-CD107a-PE-Cy™5 (H4A3, BD Bioscience, San Jose, CA, USA), anti-CD56-PE (N901, Beckman Coulter, Chaska, MN, USA), anti-CD3-APC (SK7, BD Bioscience, San Jose, CA, USA), anti-D8-FITC (SK1, Biolegend, San Diego, CA USA, USA), and IgG1κ-PE-Cy™5 (MOPC-21, BD Bioscience, San Jose, CA, USA, for isotype control) was carried out.

Gating strategies are shown in **Supplementary Material**. The results were expressed as counts in percentage (%) and absolute values (number of cells/µl) and as nMFI. Additionally, in both CTL and NK cell degranulation activity assays, the response extent was determined by calculating CD107a expression difference (delta) between baseline and stimulated (frequency and nMFI). Moreover, the increase in the response was also evaluated as [% response in the stimulated tube − (% response of the basal tube)/% response of the basal tube].

#### Peripheral Cytokine Quantification

Cytokine levels (IL-6, TNF-α, IFN-*γ*, IL-17A, IL-10, IL-8, IL-1β, and TGF-*β*1) were evaluated in plasma samples obtained from EDTA anticoagulated peripheral blood.

IL-6, TNF-α, IFN-*γ*, IL-17A, and IL-10 were assessed using a commercial cytometric bead array (CBA) kit, the CBA Human Th1/Th2/Th17 kit (BD Bioscience, San Jose, CA, USA); IL-8 and IL-1β were assessed using the CBA Human Inflammatory Cytokine Kit (BD Bioscience, San Jose, CA, USA) following the instructions of the manufacturer. The standard curve and sample concentration calculation was performed with the FCAP Array software (BD Bioscience, San Jose, CA, USA); results were expressed as pg/ml.

On the other hand, TGF-*ß*1 was determined by a commercial quantitative sandwich enzyme linked immunosorbent assay (ELISA) (Quantikine, R&D Systems, Minneapolis, MN, USA) according to the instructions of the manufacturer. The calibration curve values were adjusted to a four-parameter logistic. TGF-*ß*1 concentration was determined from the constructed standard curve and expressed as pg/ml.

### Statistical Analysis

Statistical analysis was performed using GraphPad Prism version 5.01 (GraphPad Software Inc). To compare the means between groups, Student’s t-test or ANOVA (Newman–Keuls post-tests) was performed. To determine differences between groups not normally distributed, medians were compared using the Mann–Whitney U test or Kruskal–Wallis test (Dunn’s post-tests). To determine the differences in variables measured in the same group under different conditions (for example, basal *vs.* stimulated), a paired t-test or the Wilcoxon signed range test was used, depending on whether the distribution complied with normality or not, respectively. Pearson’s correlation coefficient was used to measure the degree of association between continuous, normally distributed variables. The degree of association between non-normally distributed variables was assessed using Spearman’s non-parametric correlation. In all cases, Shapiro–Wilk test was used to evaluate normal distribution. P-values <0.05 were considered statistically significant. **Supplementary Table S3** shows the dataset analyzed during the current study.

## Results

### Characterization of Liver Microenvironment

Three components of liver pathogenesis in CHC were considered in this manuscript: liver HCV infection, the inflammatory infiltrate, and the cytokine milieu. Clinical, virological, and histological features of the patients are described in **Table 1**.

**Table 1:** Clinical, virological and histological patient features.

		Chronic HCV Patients (n = 48)
**Age (years)** median (min.–max.)		54 (32–72)
**Gender**	Male: Female	1:1
**Risk factor for HCV infection %** (n/total)	Drug abuse	18.75 (9/48)
	Transfusion	16.68 (8/48)
	Sexual	8.33 (4/48)
	Hemodialysis	2.08 (1/48)
	Occupational	2.08 (1/48)
	Tattoo	2.08 (1/48)
	Unknown	50 (24/48)
**Genotype %** (n)	1a	41.67 (20/48)
	1b	41.67 (20/48)
	2	6.25 (3/48)
	3a	6.25 (3/48)
	4	2.08 (1/48)
	ND	2.08 (1/48)
**Viral load (IU/ml)** median		1160000
(min.–max.)		(343–82,400,000)
**ALT (IU/L)** median (min.–max.)		70 (10–330).
% (n/total)	Elevated	80.85 (38/47)
**AST (IU/L)** median (min.–max.)		56 (14–296)
% (n/total)	Elevated	63.83 (30/47)
**Hepatitis^1^%** (n/total)	Minimal	2.27 (1/44)
	Mild	18.18 (8/44)
	Moderate	59.09 (26/44)
	Severe	20.46 (9/44)
**Fibrosis stage^2^%** (n/total)	F0	4.55 (2/44)
	F1	31.82 (14/44)
	F2	29.54 (13/44)
	F3	25.00 (11/44)
	F4	9.09 (4/44)
	**Advanced Fibrosis (≥3)**	34.09 (15/44)

ND, not determined; ALT, alanine aminotransferase; AST, aspartate aminotransferase; normal ALT and AST levels for adult patients were ≤40 and ≤42 IU/L, respectively when testing was done at 37°C. ^1^Hepatitis classification: minimal (HAI≤ 3), mild (HAI 4–6), moderate (HAI 7–12) and severe hepatitis (HAI>12). ^2^Fibrosis according to METAVIR. Four patients have a non-evaluable liver biopsy and therefore no information is available about liver damage.

### HCV-NS3 Detection in Liver Biopsies

**Figure 1:**
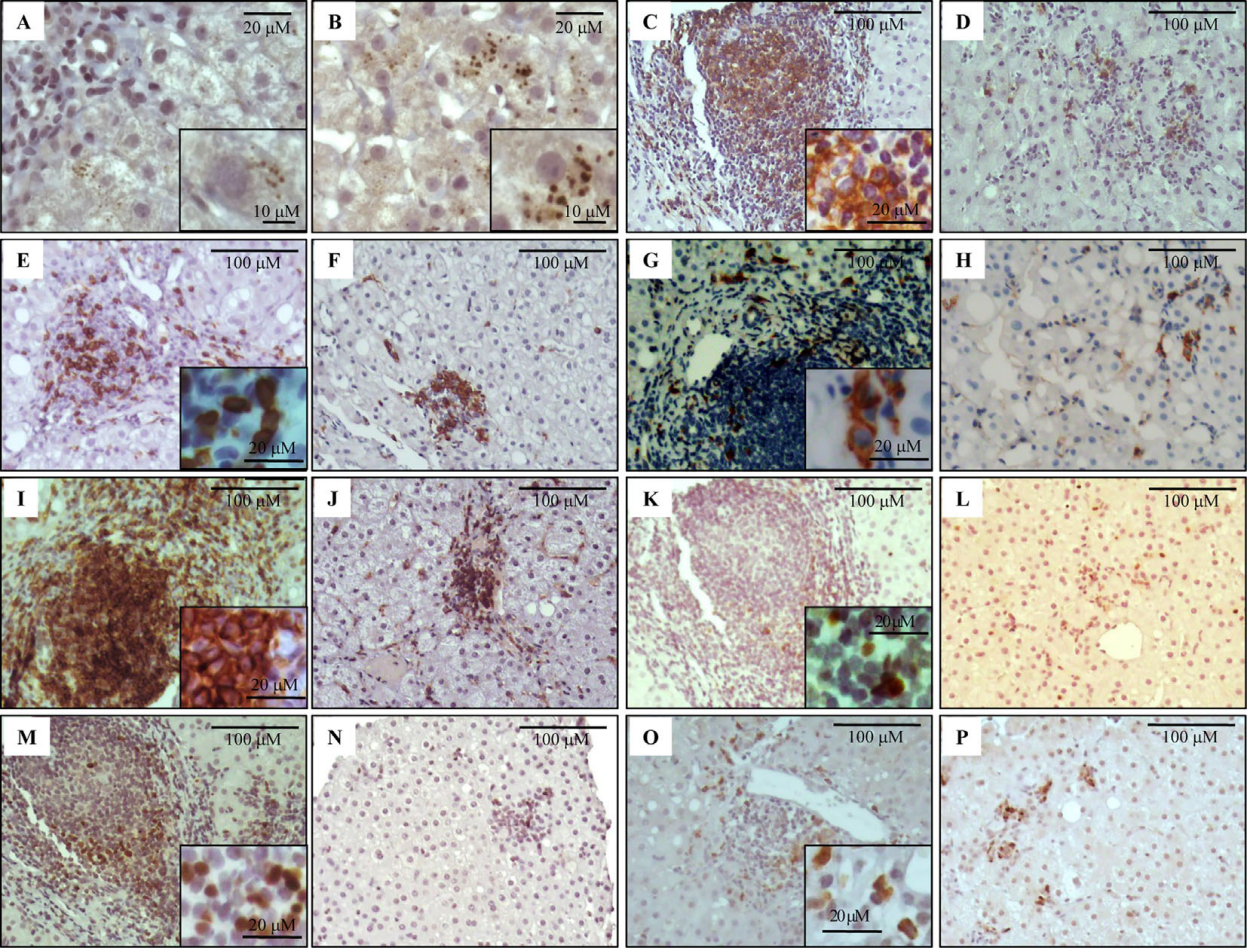
Immunostaining of NS3 and liver infiltrating lymphocyte populations on formalin-fixed paraffin embedded liver biopsies. Representative images of NS3+ hepatocytes **(A, B)**, CD20+ **(C, D)**, CD8+ **(E, F)**, CD56+ **(G, H)**, CD4+ **(I, J)**, Tbet+ **(K, L)**, Foxp3+ **(M, N)** and IL-17A+ **(O, P)** lymphocytes. Portal–periportal tract **(A, C, E, G, I, K, M, O)** and lobular area **(B, D, F, H, J, L, N, P)**.

A cytoplasmic, mainly perinuclear, NS3 immunostaining with a granular pattern of variable intensity was observed. Some cases displayed both portal–periportal (P–P) and lobular arrangement, while others showed only P–P distribution (**Figure 1**). The infected hepatocyte frequency was variable among cases [median: 0.052 (min.–max: 0.005–0.338)]. It did not display an association with liver damage (**Supplementary Figure S1A**), and no correlation with viral load or transaminases was detected (NS3 *vs.* viral load: r = −0.059, *p* = 0.765; NS3 *vs.* AST: r = 0.096, *p* = 0.602; NS3 *vs.* ALT: r = −0.011, *p* = 0.952).

### Analysis of the Inflammatory Infiltrate

Immunolabeling of B lymphocytes (CD20+), CTL (CD8+), NK cells (CD56+), Th lymphocytes (CD4+), and Th1 (Tbet+), Treg (Foxp3+) and Th17 (IL-17A+) at P–P areas was observed with scattered lymphocytes in the lobular region (**Figure 1**). Concerning P–P cell frequency, Th lymphocytes were predominant followed by CTL, B lymphocytes, and NK cells. When analyzing Th subset frequency, Th17 showed the lowest counts (**Table 2** and **Supplementary Figure S1B**). On the other hand, there was predominance of CTL and Th1 at the lobular area, together with absence of B lymphocytes and NK cells (**Table 2** and **Supplementary Figure S1B**).

**Table 2:** Quantification of liver cell populations.

Cell population	Portal–periportal area	Lobular area
**Th**	0.66 (0.04–0.85)	0.20 (0–5.78)
**CTL**	0.48 (0.15–0.75)	2.00 (0–15.00)
**B**	0.25 (0–0.62)	0 (0–2.20)
**NK**	0.01 (0–0.09)	0 (0–0.50)
**Th1**	0.09 (0–0.39)	1.20 (0–12.57)
**Treg**	0.11 (0–0.29)	0.30 (0–1.90)
**Th17**	0.07 (0.01–0.26)	0.20 (0–1.50)

Results are expressed as: a) portal–periportal frequencies: immunostained/total cells; b) lobular frequencies: immunostained cells/field (×400).

**Figure 2:**
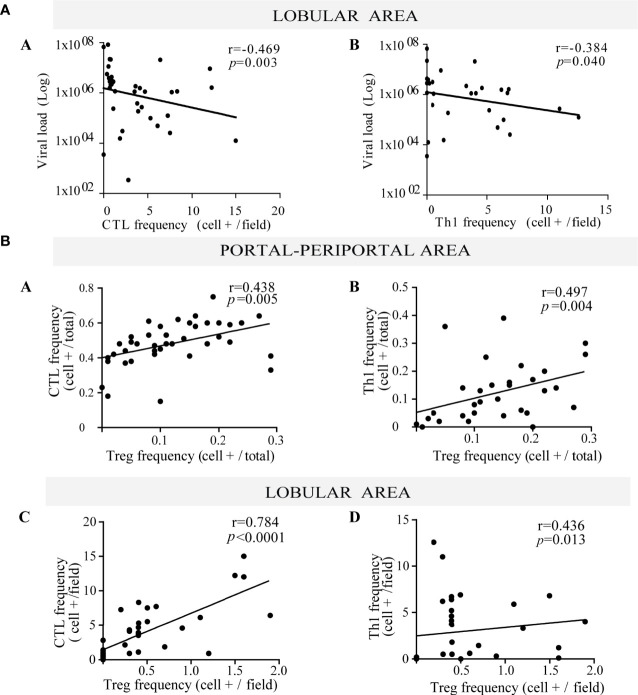
**(A)** Relationship between viral load and frequency of both lobular CTL and Th1. Correlation between viral load and the frequency of lobular CTL (a) and Th1 (b). **(B)** Relationship between Treg lymphocytes with both CTL and Th1. Correlation between the frequency of Treg with CTL (a) and Th1 (b) at portal-periportal area. Correlation between the frequency of Treg with CTL (c) and Th1 (d) at lobular area. Spearman’s nonparametric correlation was used to compare these data sets.

CTL and Th1 cells are well known components of the antiviral immune response. In this cohort despite their lobular predominance, they did not correlate with the number of infected hepatocytes, but they disclosed a negative correlation with viral load (r = −0.469, *p* = 0.003 and r = −0.384, *p* = 0.040; respectively) (**Figure 2A**). Since viral load mirrored HCV liver replication, this could indirectly suggest CTL and Th1 immune control of the liver process.

When evaluating the interrelation among different hepatic immune cells, Treg depicted a positive correlation with CTL and Th1 at the P–P area (CTL *vs.* Treg: r = 0.438, *p* = 0.005; Th1 *vs.* Treg: r = 0.497, *p* = 0.004) and lobular area (CTL *vs.* Treg: r = 0.784, *p* < 0.0001; Th1 *vs.* Treg: r = 0.436, *p* = 0.013) (**Figure 2B**), which would indicate that Treg may control effector lymphocytes (CTL and Th1).

**Figure 3:**
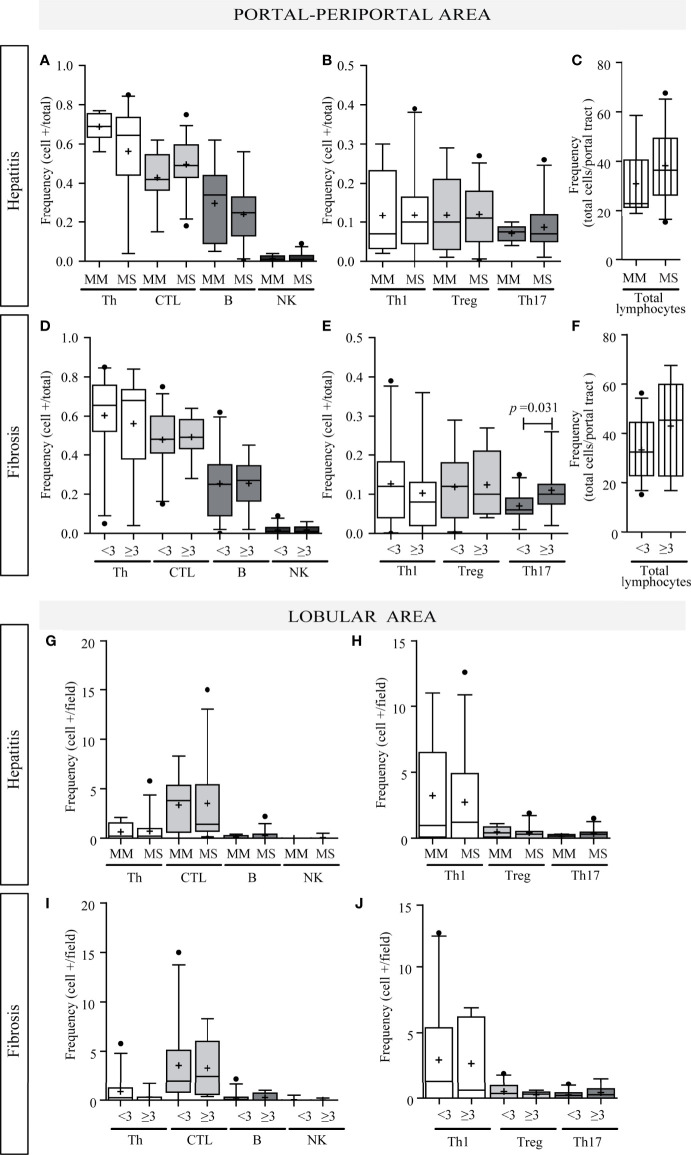
Relationship between intrahepatic infiltrate and liver damage. Portal–periportal cell population frequencies related to hepatitis **(A–C)** and fibrosis severity **(D–F)**. Lobular cell population frequencies related to hepatitis **(G, H)** and fibrosis severity **(I, J)**. Th, CTL, B lymphocytes and NK cell **(A, D, G, I)**; Th subpopulation **(B, E, H, J)**; and total portal lymphocytes **(C, F)**. MM, minimal–mild; MS, moderate–severe hepatitis. Advanced fibrosis (F≥3) according to METAVIR. The results are depicted in box plots. Horizontal lines within boxes indicate medians. Horizontal lines outside the boxes represent the 5 and 95 percentiles. Mean is indicated as +. Mann–Whitney U test was used to compare all data sets, except for portal–periportal CTL, B, Th1, and Treg cells *vs.* hepatitis severity, and CTL and B cell *vs.* fibrosis severity for which Student’s t-test was applied.

Concerning liver damage (**Figures 3A–J**), P–P Th17 was the only lymphocyte subset that seemed to be related to liver damage; in fact Th17 lymphocytes were associated with advanced fibrosis (*p* = 0.0312) (**Figure 3E**). Total P–P lymphocytes correlated with inflammatory activity (r = 0.376, *p* = 0.014) (**Supplementary Figure S1C-a**) and depicted an augmented profile in severe fibrosis (**Figure 3F**) which was consistent with the association between inflammatory activity and fibrosis severity (*p* = 0.04) (**Supplementary Figure S1 C-b**). Besides Th17 and CTL correlated with transaminase levels (CTL *vs.* AST: r = 0.412, *p* = 0.008; CTL *vs.* ALT: r = 0.403, *p* = 0.009; Th17 *vs.* AST: r = 0.594, *p* < 0.0001) (**Supplementary Figure S1D**).

### Quantification of Liver Cytokine Expression

**Figure 4:**
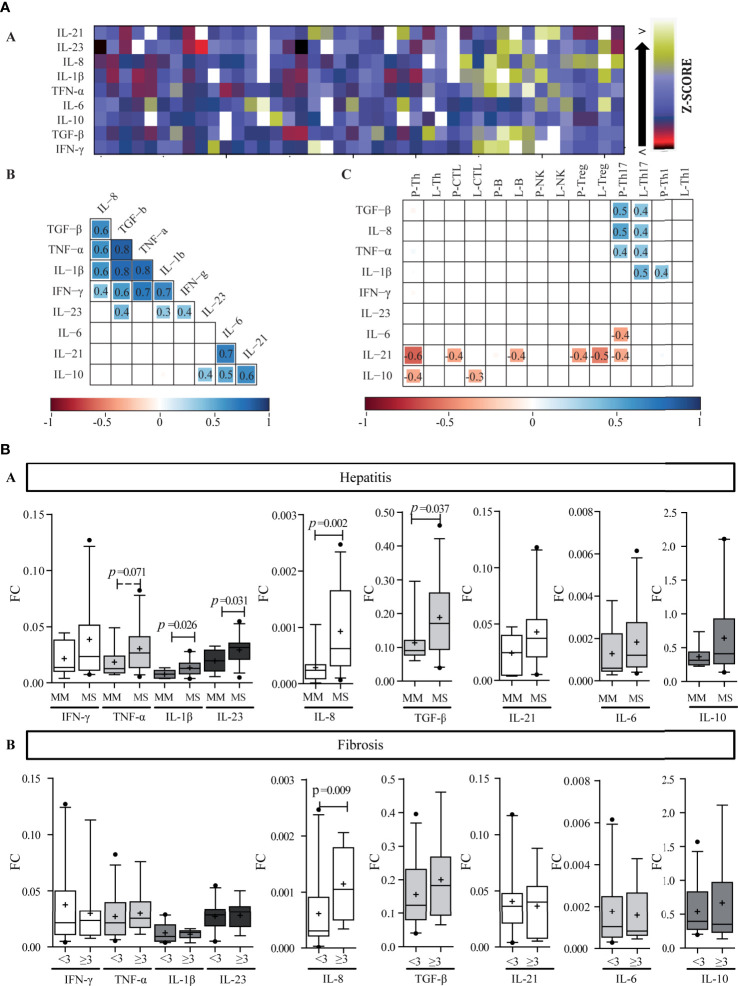
Liver cytokine expression level. **(A)** Heat map of liver cytokine expression level (a). Each row corresponds to the cytokine indicated at the top, and each column represents one case. The data are normalized, and the results are expressed as a Z-score (Xi-/SD), which is assigned a color scale that goes from black (lowest values) to yellow (highest values). The white boxes correspond to the cases without data. Correlation matrix of cytokines (b) and correlation matrix between infiltrate cell populations and intrahepatic cytokine levels (c). (b, c) are schematic representation: the numbers inside the boxes indicate the Spearman correlation coefficients (r) only for those pairs of cytokines that show statistical significance (*p* < 0.05). The squares inside the boxes graphically represent the value of r: the light blue color indicates r values between 0 and 1 (positive correlation), and the red color indicates values between −1 and 0 (negative correlation), while its size increases as r increases towards values close to |1|. **-**P, portal–periportal; -L, lobular. Panel **(B)** Relationship between intrahepatic cytokine levels and liver damage. Expression levels of intrahepatic cytokines in relation to hepatitis (a) and fibrosis (b) severity. MM, minimal–mild; MS, moderate–severe hepatitis. Advanced fibrosis (F≥3) according to METAVIR. The results are depicted in box plots. Horizontal lines within boxes indicate medians. Horizontal lines outside the boxes represent the 5 and 95 percentiles. Mean is indicated as +. FC, fold change. Mann–Whitney U test was used to compare all data sets, except for IL-1β and IL-23 *vs.* hepatitis severity for which Student’s t-test was applied.

Pro-inflammatory cytokines TNF-α, IL-23, IFN-*γ*, IL-1β, IL-6, IL-8, IL-17A, and IL-21, as well as anti-inflammatory IL-10 and TGF-*β* expression were quantified in fresh liver biopsy samples. The relative expression of different cytokines was variable (**Figure 4A-a**). Interestingly, IL-17A was undetectable in most cases which agreed with Th17 low frequency evaluated by immunohistochemistry.

Two correlation matrixes were performed, the first one linked cytokine to each other (**Figures 4A, B**) and the second one related each cytokine with each immune cell population (**Figure 4 A-c**). Interestingly, among the cytokines two clear groups were delineated: Group 1 (G1), included IFN-*γ*, TNF-α, IL-1β, IL-8, and TGF-*β*, and Group 2 (G2) included IL-6, IL-21, and IL-10. IL-23 presented a particular behavior given that it correlated with TGF-*β*, IL-1β, and IFN-*γ* from G1, and IL-10 from G2. When considering the ability of the hepatic milieu to condition the permanence of the Th subpopulations or to influence their differentiation and plasticity, TGF-*β*, IL-21, IL-β, and IL-6 were quantitatively related to Th17 frequency. These cytokines had an opposite behavior, namely TGF-*β* and IL-1β depicted direct correlation with Th17, while IL-6 and IL-21 an inverse one (**Figure 4A-c**). Of note Tregs showed no relationship with TGF-*β*.

Regarding the relation between cytokines and their producer cells, IFN-*γ* and TNF-α showed no correlation with CTL, Th1, or NK cells, while Treg did not correlate with TGF-*β* and IL-10 expression. Strikingly, IL-21 was inversely correlated with Th17 frequency; nevertheless, it should be considered that other cell subsets could also produce IL-21. On the other hand, it ought to be noted that the inverse correlation of IL-21 with Th, CTL, B, and Treg frequency as well as the correlation of IL-10 with Th and CTL could indicate a negative effect of these cytokines on the inflammatory infiltrate (**Figure 4A-c**). Finally, infected hepatocyte frequency inversely correlated with IL-10 and IL-21 expression levels (r = −0.495, *p* = 0.006; r = −0.446, *p* = 0.023, respectively) indicating their possible role on viral control (**Supplementary Figure S1E**).

When analyzing the relation of cytokines and liver damage, a trend to higher values in those cases with more severe hepatitis was observed, but it turned out to be significant only for IL-1β (*p* = 0.026), IL-23 (*p* = 0.031), IL-8 (*p* = 0.002), and TGF-β (*p* = 0.037); while concerning fibrosis severity only IL-8 was associated with advanced fibrosis (*p* = 0.009) (**Figure 4B**). Moreover, IL-8 also displayed a positive correlation with P–P and lobular Th17 frequency (**Figure 4A-c**), which, as mentioned above, depicted an association with fibrosis severity too (**Figure 3E**). Finally, TGF-*β*, TNF-α, IL-1β, and IL-8 displayed positive correlations with transaminase levels [AST *vs*, a) TGF-β: r = 0.554, *p* = 0.0002; b) TNF-α: r = 0.492, *p* = 0.001; c) IL-1β: r = 0.444, *p* = 0.005; d) IL-8: r = 0.439, *p* = 0.006; and ALT *vs*: e) TGF-β: r = 0.318, *p* = 0.043; f) TNF-α: r = 0.355, *p* = 0.020].

### Evaluation of the Peripheral Immune Response

#### Quantification of Peripheral Lymphocytes Frequency

The comparative analysis of T and B lymphocyte populations did not reveal significant differences between donors and patients (**Table 3**) and depicted the same frequency proportion as described for portal hepatic infiltrate (**Supplementary Figure S2A**). In contrast, NK cells showed a significant decrease in absolute values (*p* = 0.008) in patients accompanied by a decreased in NK *dim* (*p* = 0.02 percentage and *p* = 0.018 absolute value) along with an increase in NK *bright* (*p* = 0.025 percentage value) (**Table 3**).

**Table 3:** Quantification of peripheral cell populations.

Cell population	Percentage value (%)		*p-*value	Absolute value (cells/µl)		*p-*value
	Donor	HCV		Donor	HCV	
**Th**	46.00	44.50	0.606	940.4	822.8	0.185
	(29.00–51.0)	(18.00–68.00)		(576.0–2144.0)	(187.9–2747.0)	
**CTL**	26.00	26.00	0.957	664.6	537.6	0.101
	(19.00–37.00)	(11.00–63.00)		(332.8–1169.0)	(181.2–1017.0)	
**B**	10.50	12.00	0.148	237.0	255.8	0.531
	(7.00–16.80)	(1.30–31.20)		(115.2–506.5)	(27.81–601.7)	
**NK**	13.55	9.00	0.056	346.0	201.0	0.008
	(5.60–32.00)	(1.30–31.00)		(94.60–755.0)	(35.02–626.6)	
**NK *dim***	97.80	91.30	0.025	335.1	129.0	0.018
	(89.30–99.10)	(69.60–97.50)		(84.50–480.8)	(52.47–376.0)	
**NK *bright***	2.20	8.00	0.025	9.80	15.45	0.165
	(0.90–10.70)	(3.10–31.10)		(2.40–24.30)	(2.84–43.07)	
**Th1**	3.50	3.75	0.380	33.97	28.85	0.586
	(0.39–17.80)	(0.77–15.30)		(2.23–136.4)	(5.57–167.0)	
**Treg**	4.84	4.45	0.687	48.50	35.60	0.064
	(1.65–8.33)	(1.82–9.79)		(30.34–131.2)	(14.40–181.6)	
**Th17**	0.51	0.38	0.078	4.12	3.28	0.128
	(0.12–1.90)	(0.03–0.94)		(1.60–19.99)	(0.32–13.83)	

The results are expressed as median (min.-max.).

As to liver damage, none of the peripheral lymphocyte population percentages disclosed differences with respect to hepatitis or fibrosis severity (**Supplementary Figure S2B**). The same was observed in the analysis of absolute and nMFI values.

#### Peripheral T Lymphocyte Differentiation and Functional Characterization of NK Cells and CTLs

**Figure 5:**
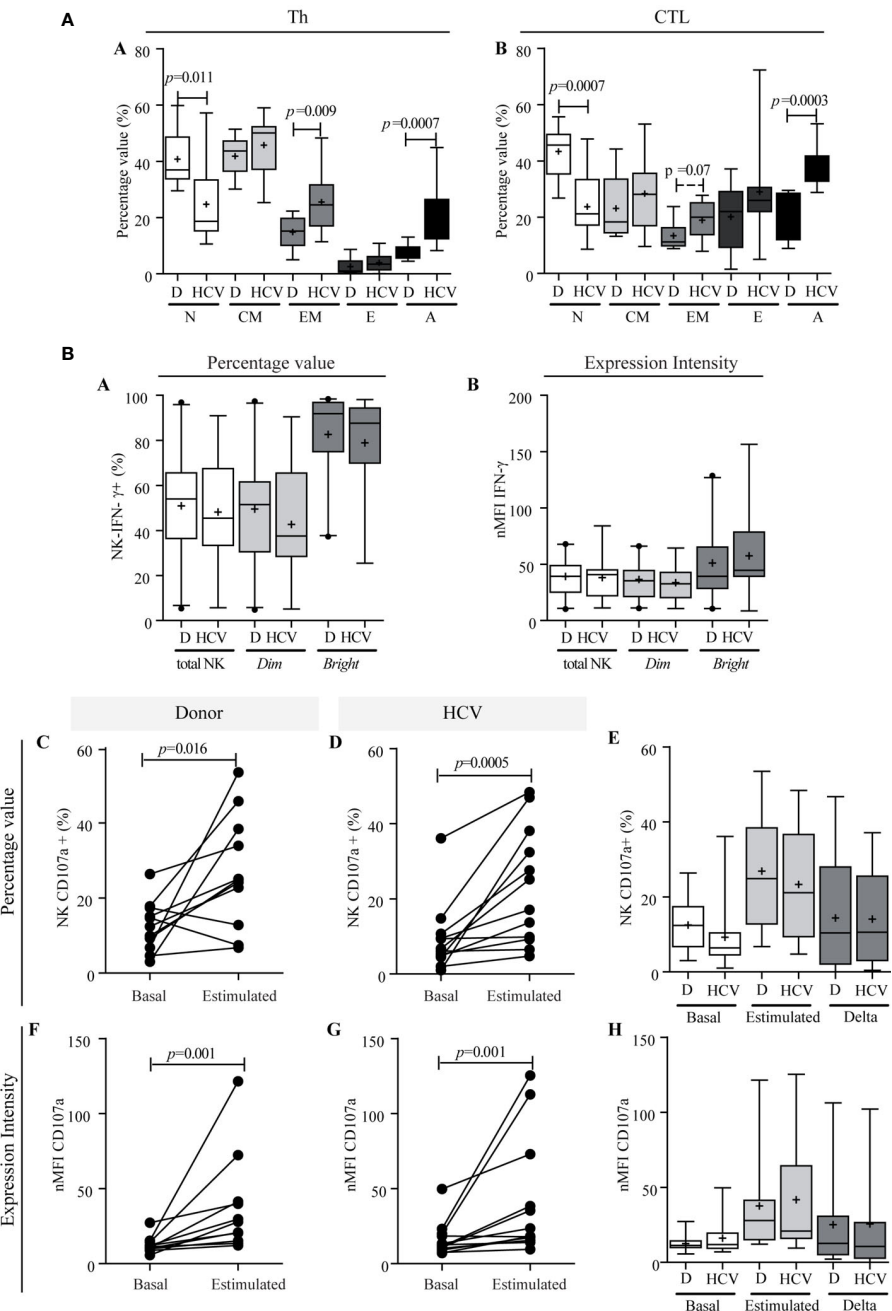
T lymphocyte differentiation and functional characterization of NK cells. **(A)** Comparison of lymphocytes differentiation stages between donors and patients. Th (a) and CTL (b). Results are expressed as percentage values. N, naïve; CM, central memory; EM, effector memory; E, effector; A, activated. Student’s t-test was used to compare all data sets except for naïve Th and activated Th, and effector CTL for which Mann–Whitney U test was applied. Panel **(B)** IFN-*γ* production capacity (a, b) and degranulation activity of NK cells (c–h). IFN-*γ* production of NK cells in patients and donors (a, b). Percentage values (a), and intensity of expression (b). CD107a expression in total NK cells in percentage values (c–e) and in intensity of expression (f–h). Comparison of the response between basal conditions and before the stimulus per case in donors (c and f) and in patients (d, g). Comparison of basal CD107a expression in total NK cells, stimulated and magnitude of response (delta) between donor *vs.* patient (e and h). Results are expressed as percentage values (c–e) and intensity of expression (f–h). D, donor. When it corresponds, the results are depicted in box plots. Horizontal lines within boxes indicate medians. Horizontal lines outside the boxes represent the 5 and 95 percentiles. Mean is indicated as +. Mann–Whitney U test (a, b, e, h) and Wilcoxon test (c, d, f, g) were used to compare all data sets. *The analysis of absolute values displayed similar results to the percentage values, but it is not shown to simplify their visualization*.

The distribution of naïve, central memory, effector memory, effectors and activated CTL and Th lymphocytes was determined. As shown in **Figure 5A**, a decrease in the frequency of both naïve Th and CTL (*p* = 0.011 and *p* = 0.0007, respectively) and an increase of activated lymphocytes (*p* = 0.0007 and *p* = 0.0003, respectively) were observed in CHC patients. In turn, a trend to higher frequency of effector memory CTL (*p* = 0.07) and Th (*p* = 0.009) was demonstrated (**Figure 5A**).

With regard to NK functionality, IFN-*γ* production was similar between patients and donors [45.50% (min.–max: 5.74–91.00) *vs.* 54.0% (min.–max: 5.38–96.90), respectively], and the same applied to NK subsets, namely NK *dim* [patients: 37.55% (min.–max.: 5.09–90.59%) *vs.* donors: 51.50% (min.–max: 4.75–97.40%)], and NK *bright* [patients: 87.65% (min.–max: 25.50–98.10%) *vs.* donors: 91.90% (min.–max: 37.30–98.40%)] (**Figure 5B-a**). The nMFI analysis demonstrated similar cytokine production capacity in patients and donors both in total NK and in its subpopulations (**Figure 5B-b**). The NK degranulation activity showed a similar significant response against stimulus in patients and donors (percentage value: donor *p* = 0.016, HCV *p* = 0.0005; nMFI: donor *p* = 0.001, HCV: *p* = 0.001) (**Figure 5B-c, d, f, g**). The median of frequency increase was 1.49-fold (min.–max: 0.05–37.10) in patients *vs.* 0.84 (min.–max: 0.50–11, 70) in donors, while the median increase in CD107a expression intensity (nMFI) was 0.85 times (min.−max: 0.0–4.70) in patients *vs.* 1.0 times (min.–max: 0.20–7.0) in donors. The response magnitude (delta) in the NK degranulation activity assay was similar between patients and donors (**Figure 5B-e, h** **)**. In summary, these observations indicated that NK functionality was not impaired in HCV infection, since IFN-*γ* production and degranulation activity were not altered.

In relation to liver damage, NK cells IFN-*γ* production and degranulation activity showed no association with either fibrosis or hepatitis (**Supplementary Figure S3**). Nonetheless, the lowest basal degranulation percentages were found in those cases with advanced fibrosis, but, when considering the nMFI, an inverse profile was observed, with significantly higher values in NK (*p* = 0.016) and NK *dim* (*p* = 0.021), conceivably as a compensating mechanism (**Supplementary Figure S3B-j, k**).

**Figure 6:**
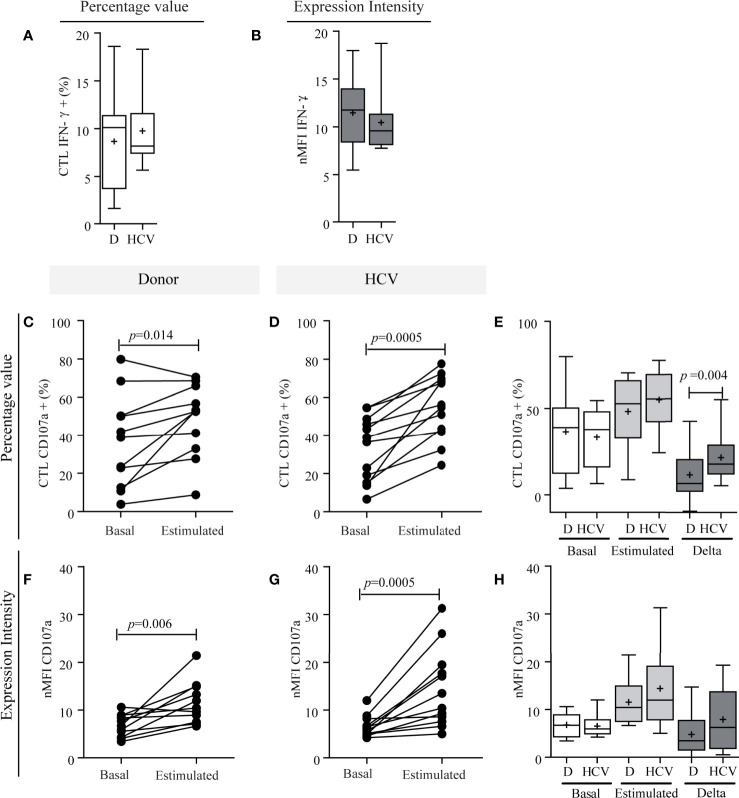
Functional characterization of CTLs. IFN-*γ* production capacity **(A, B)** and degranulation activity of CTLs **(C–H**). IFN-*γ* production of CTLs in patients and donors **(A, B)**. Percentage values **(A)** and intensity of expression **(B)**. CD107a expression in CTLs in percentage values **(C–E)** and in intensity of expression **(F–H)**. Comparison of the response between basal conditions and before the stimulus per case in donors **(C, F)** and in patients **(D, G)**. Comparison of CD107a basal expression in CTLs, stimulated and magnitude of response (delta) between donor *vs.* patient **(E, H)**. Results are expressed as percentage values **(C–E)** and intensity of expression **(F–H)**. D, donor. When it corresponds, the results are depicted in box plots. Horizontal lines within boxes indicate medians. Horizontal lines outside the boxes represent the 5 and 95 percentiles. Mean is indicated as +. Student’s t-test **(A, E)**, Wilcoxon test **(C, D, G)**, paired t-test **(F)** and Mann–Whitney U test **(B, H)** were used to compare different data sets. *The analysis of absolute values displayed similar results to the percentage values, but it is not shown to simplify their visualization*.

Concerning CTL functionality, IFN-*γ* production showed no differences between patients and donors [HCV: 8.17% (min.−max.: 5.66−18.30) *vs.* donors of 10.10% (min.−max: 1.65−18.60) in percentage value; HCV: 9.58 (7.77–18.71) *vs.* donors: 11.75 (5.49–18.96) in nMFI (**Figures 6A, B**)]. Related to degranulation activity, the basal expression of CD107a showed no significant differences between patients and donors (**Figures 6E, H**), while the stimulus triggered a significant degranulation increase in both groups (percentage value: HCV, *p* = 0.0005; donors, *p* = 0.014; nMFI: HCV, *p* = 0.0005; donor, *p* = 0.006) (**Figures 6C, D, F, G**). The median increase in percentage was of 0.60-fold (min.−max: 0.15−4.00) in patients and 0.26-fold (min.−max.: −0.12 to 397) in the donors, and in nMFI the median increase was 1.20-fold (min.−max: 0.10–3.0) in patients and 0.70-fold (min.−max.: −0.10 to 2.20) in the donors. The response magnitude (delta) in percentage values was significantly elevated in patients (*p* = 0.004) (**Figure 6E**). These results would indicate that CTL functionality was not impaired in HCV infection since IFN-*γ* production and the ability to degranulate in response to the stimulus were not altered, but HCV patient demonstrated a greater magnitude of degranulation activity in response to the stimulus.

Finally, in relation to liver damage, CTL IFN-*γ* production in response to the stimulus did not show any relationship (**Supplementary Figure S4A**). Regarding degranulation activity in response to the stimulus, no differences were observed in the percentage of CTL CD107a+ according to fibrosis severity (**Supplementary Figure S4B-c**), but nMFI was significantly high in cases with more severe fibrosis (*p* = 0.008) (**Supplementary Figure S4B-d**). In turn, delta response was greater both in percentage and nMFI values in patients with higher severity of fibrosis (*p* = 0.016; *p* = 0.008, respectively) (**Supplementary Figure S4B-c, d**).

### Quantification of Peripheral Cytokines

**Figure 7:**
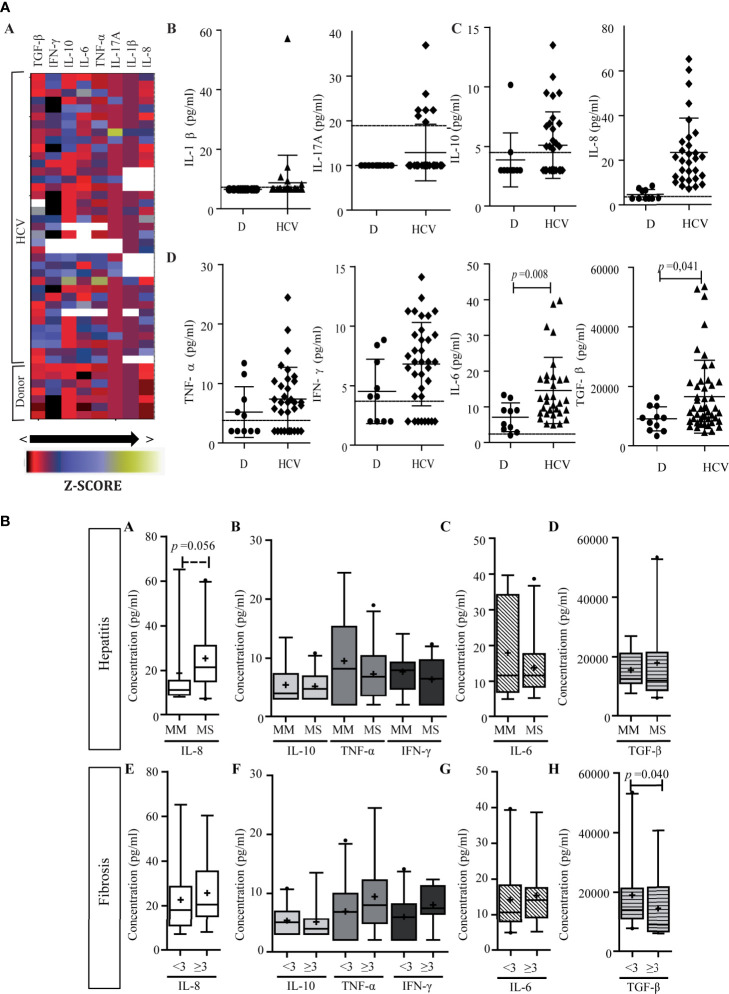
Peripheral cytokine level. **(A)** Heat map of peripheral cytokine expression levels (a) and comparison of circulating cytokine levels between patients and donors (b–d). (a) Each row corresponds to the cytokine indicated at the top, and each column represents one case. The data are normalized, and the results are expressed as a Z-score (Xi-/SD), which is assigned a color scale from black (lowest values) to yellow (highest values). The white boxes correspond to the cases without data. (b–d) Cytokine levels in patients and donors: IL-1β and IL-17A (b), IL-10 and IL-8 (c), and TNF-α, IFN-*γ*, IL-6, and TGF-*β* (d). D donor. Results are plotted on dot diagrams, the middle horizontal line indicates the mean, and the outer horizontal lines represent the SD. Mann–Whitney U test was used to compare data sets. **(B)** Relationship between circulating cytokine levels and liver damage. Circulating levels of cytokines in relation to hepatitis (a–d) and fibrosis (e–h) severity. MM, minimal–mild; MS, moderate–severe hepatitis. Advanced fibrosis (F≥3) according to METAVIR. The results are depicted in box plots. Horizontal lines within boxes indicate medians. Horizontal lines outside the boxes represent the 5 and 95 percentiles. Mean is indicated as +. Mann–Whitney U test was used to compare data sets.

The heat map (**Figure 7A-a**) pointed out that cytokine levels showed a large dispersion among patients. When compared with donors, three cytokine groups may be distinguished: a) those with values below the detection limit in donors and most patients (IL-1β and IL-17A) (**Figure 7A-b**); b) those with values below the detection limit in most donors, but increased in patients (IL-10 and IL-8) (**Figure 7A-c**); and c) those with values above the detection limit in both groups with a particular trend to higher values in patients (TNF-α, IFN-*γ*, IL-6, and TGF-*β*) (**Figure 7A-d**). In the last group it is important to highlight that IL-6 and TGF-β increased significantly (*p* = 0.008 and *p* = 0.041; respectively).

Finally, cytokine levels did not display correlation between intrahepatic and peripheral compartments. In relation to liver damage, plasma IL-8 showed a trend of association with hepatitis severity (*p* = 0.056) (**Figure 7B-a)**. On the other hand, circulating TGF-*β* higher levels were observed in those cases with lower fibrosis severity (*p* = 0.040) (**Figures 7B-h**).

**Supplementary Figure S5** showed a schematic representation of the results.

## Discussion

The immune response has a dual role during the course of HCV infection, so in this manuscript we intended to evaluate the potential interplay between the virus and the liver immune microenvironment in CHC. Both intrahepatic and peripheral immune response components were analyzed in relation to virological and histological parameters to assess its possible role in the pathogenesis. To determine if the inflammatory process was localized or if immunological alterations occurred at the systemic level, the results of the evaluated parameters in both compartments were integrated.

We demonstrated that most liver infection cases displayed NS3 expression, but in a very low number of hepatocytes. The scarce number of HCV+ hepatocytes observed reinforced previous findings of our group (Valva et al., 2014) and agreed with other authors that also observed low frequencies of infected hepatocytes using different labeling techniques (Sansonno et al., 2004; Liang et al., 2009; Wieland et al., 2014). According to this observation, liver parenchymal affection seemed not to be very extensive since a high proportion of the hepatocytes remained uninfected. In agreement with Liang et al., it could be hypothesized that the immune response participates in liver infection control (Liang et al., 2009). In this regard, our previous findings support this assumption since pediatric patients who showed a less preponderant hepatic immune response than adults displayed the highest frequencies of infected hepatocytes (Valva et al., 2014). Consistent with this hypothesis, CTL and Th1 lymphocytes, central components of the antiviral immune response (Mengshol et al., 2007), displayed a lobular predominance and negative correlation with viral load, which could indicate a possible control of liver viral replication. However, a lack of correlation between liver CTL frequency and the number of infected hepatocytes was observed which may have two different explanations. First, considering that viral replication is a dynamic process, the detection of viral antigens indicates that the cells are infected but does not take into account how many viral particles are contained in each cell. Second, it could be possible that CTLs are not all virus-specific, so they could also eliminate uninfected hepatocytes, which is known as ‘bystander killing’ (Spengler and Nattermann, 2007). The whole CTL population might influence liver damage generation, which could be suggested by the positive correlation between CTL frequency and transaminase levels. Therefore, although the immune response participates in viral replication control, it seems to be also implicated in liver damage generation. In the present cohort, intrahepatic inflammatory cytokines, especially IL-1β, IL-8, and TGF-β, presented a profile tending to higher values in cases with most severe hepatitis, together with positive correlations of TGF-β, TNF-α, IL-1β, and IL-8 with transaminase levels denoting the idea of the involvement of the immune response in liver damage. Furthermore, the total portal lymphocyte count, which accounts for the magnitude of the infiltrates, correlated with the inflammatory activity, which in turn showed significantly higher values in cases with advanced fibrosis. Consequently, these observations all together support the idea that the immune response is involved in generating liver damage and particularly that the inflammation would contribute to liver fibrogenesis.

Regarding the possible role of each studied lymphocyte population in relation to liver damage, only Th17 lymphocytes seemed to be involved in CHC pathogenesis. In spite of its known inflammatory potential and its participation in various autoimmune and liver pathologies (Paquissi, 2017; Veldhoen, 2017), the role of Th17 lymphocytes in CHC pathogenesis has not yet been fully clarified. In this series we described a low frequency of intrahepatic Th17 lymphocytes compared to the other Th subpopulations evaluated; however, portal Th17 lymphocytes were associated with biochemical and histological parameters of liver damage, namely plasma AST levels and increased severity of fibrosis. Therefore, although Th17 cells were less frequent than the others, just their presence at the right time of the development of the chronic process might mediate mechanisms that may lead to or contribute to fibrogenesis. Additionally, intrahepatic Th17 lymphocyte frequency correlated with IL-8 liver expression, and both of them displayed an association with fibrosis severity, suggesting possible involvement in liver fibrogenesis which was also described in *in vitro* studies by other authors. In this sense, it has been described that the main function of both IL-17A and IL-17F is the recruitment of neutrophils and macrophages (Veldhoen, 2017). It has been demonstrated *in vitro* that these cytokines are capable of inducing the secretion of chemokines such as CCL7 and CXCL8 (IL-8) (Veldhoen, 2017). In turn, IL-8 has been widely related to various liver pathologies, and it is postulated that, in addition to promoting the recruitment of neutrophils, it would contribute to the activation of stellate cells, and consequently to liver fibrogenesis (Zimmermann et al., 2011; Xu et al., 2014). Regarding the role of Th17 cells at the peripheral compartment, they were also underrepresented; likewise IL-17 was undetectable in most studied samples as described by other authors (Foster et al., 2012; Sousa et al., 2012). Besides, their peripheral frequency did not display an association with liver damage parameters, supporting the idea of a localized contribution to the pathogenesis.

As previously mentioned, Th17 lymphocytes represents the Treg counterpart since they share differentiation process mediators and these Th subsets could be also repolarized (DuPage and Bluestone, 2016). In the studied cohort, TGF-β, IL-21, IL-6, and IL-1β combined with hepatic expression favor a Th17 scenario, although the individual role of each cytokine as well as their effect on the Treg lymphocytes remains to be elucidated. This is important since the hepatic cytokine milieu could generate a bias towards a certain lymphocyte profile, which in turn favors or controls liver damage, hence delineating the course of the disease.

In relation to Treg lymphocytes, although its role is still controversial (Claassen et al., 2010; Sturm et al., 2010), their significant presence, especially at portal tracts, in CHC liver biopsies could denote their involvement in immune response modulation. The correlations between Treg with both CTL and Th1, at portal and lobular areas, could suggest that there could be an increase recruitment or differentiation towards a Treg profile induced by the inflammatory state established in the liver; their presence could favor the control of effector lymphocytes (CTL and Th1). In this sense, the lack of association between CTL and Th1 with hepatitis or fibrosis severity could be interpreted as a consequence of Treg modulation by inhibiting Th1 and CTL actions and in turn preventing liver damage generation. Further, supporting this theory, the negative correlations between hepatic IL-10 expression and both portal Th and lobular CTL would denote their participation in the control of inflammation (Barjon et al., 2015; DuPage and Bluestone, 2016). Interestingly, this modulation is not observed in the peripheral blood compartment, reinforcing the idea of an action that occurred at the infection site but not in a generalized manner.

In a comprehensive approach of the liver microenvironment, it is important to consider the interplay between intrahepatic cytokines and the hepatic producer lymphocyte populations. First, the absence of a quantitative relationship between IFN-**γ** or TNF-α expression levels and the CTL or Th1 frequency could indicate that these cells may have variable activation status and consequently an uneven level of cytokine production. Furthermore, it is important to highlight that during the chronic stage of the infection, various authors have reported an exhaustion of both CTL and Th, so their functions could be compromised (Kroy et al., 2014). However, it may be also possible that the observed discrepancy is based on the fact that CTL or Th1 is not the main source of IFN-**γ** or TNF-α production in the liver. In addition, the absence of correlation between IL-10 and TGF-β liver expression and Treg frequency could be due to diverse activation status in cases with different liver damage severity or due to the contribution of other cell types not evaluated in this study, such as macrophages and Kupffer cells (Hartling et al., 2016).

At last, it is interesting to discuss the intrahepatic expression of the pleiotropic cytokine IL-21, mainly produced by Th17 and Th follicular (Thf) lymphocytes (Spolski and Leonard, 2014). Based on peripheral blood assays, it was proposed that IL-21 in CHC would be protective since it stimulated CTL activity thereby promoting viral elimination and limiting liver damage (Kared et al., 2013; Cachem et al., 2017). However, there are no previous studies evaluating intrahepatic IL-21 in CHC. In this cohort an inverse relationship between IL-21 liver expression and frequency of infected hepatocytes was described, which would support the hypothesis of the protective role of IL-21. In turn, IL-21 showed negative correlations with most of the evaluated lymphocyte populations, namely B lymphocytes, Th, CTL, Treg, and Th17 indicating that it participated in some way by limiting inflammation. Likewise, IL-10 would also participate in infection control given its inverse correlation with infected hepatocytes, although the underlying mechanism is not clear from the global analysis of our results.

Even though CHC is mainly a liver disease, adult patients have also been reported to present extrahepatic manifestations, which could be a consequence of immunological disorders. Advanced CHC could be accompanied by the presence of cryoglobulins that cause renal, dermatological, hematological, and rheumatic complications (Tang et al., 2016). Interestingly, in the peripheral blood assays performed in this series, no differences in T and B lymphocyte counts were observed between patients and non-infected donors, which would indicate at first glance that the infection did not alter the distribution of lymphoid populations. However, the ability of the virus to infect lymphocytes (Gismondi et al., 2013) and the persistent antigenic stimulation might cause alterations in T lymphocyte differentiation status (Kuniholm et al., 2014). In this sense, a decrease in both Th and CTL naïve lymphocytes and an increase in EM phenotype and activated lymphocytes (DR+) were described in our cohort. Yet, if it is considered that the number of peripheral HCV-specific lymphocytes is extremely low (Alanio et al., 2015), this alteration in the distribution could be a consequence of a non-specific activation. Hence, in accordance with Alanio et al., one plausible explanation could be that peripheral lymphocytes would have a lower threshold for TCR activation due to HCV persistent stimulation which generates hyperactivation of non-HCV specific naïve lymphocytes (Alanio et al., 2015). These alterations would have implications in the immune response not only against HCV, but also against other pathogens or vaccines (Burke Schinkel et al., 2016) and could even be related to the presence of extrahepatic manifestations associated with chronic HCV infection (Alanio et al., 2015). To reinforce activated peripheral status observed, the evaluation of circulating cytokines in this series showed an altered profile compared to non-infected donors, with an increase in both pro- and anti-inflammatory cytokines that could also condition the establishment of an adequate immune response, even against other stimuli. Although peripheral immune response seemed to be altered, neither lymphocyte population frequency nor cytokine levels displayed an association with liver damage parameters. The only exception was the association of TGF-β with less severe fibrosis stages in CHC patients, which was previously described by our group (Valva et al., 2011). TGF-β exerted fibrogenic effects on stellate cells as well as modulatory effects on the immune response. Since fibrogenesis is considered a long process and fibrosis the final picture, a higher level of TGF-*ß* in lower liver fibrosis stages may reflect fibrogenesis rather than fibrosis (Nelson et al., 1997; Soliman et al., 2010).

To deepen understanding of the peripheral CTL role, given that many authors have described a dysfunctional IFN-**γ** production in HCV-specific CTLs (Spangenberg et al., 2005; Penna et al., 2007; Saha et al., 2013), IFN-**γ** secretion activity was explored, but no differences between HCV patients and non-infected donors arose, and no relation with liver damage severity was evidenced, perhaps due to the assessment of the total CTL instead of the HCV-specific CTL IFN-**γ** secretion. CTL degranulation activity did not seem to be impaired in HCV patients; moreover, the response (as the delta) was even greater in patients. In addition, this difference was accentuated in those cases with more severe fibrosis. Therefore, the inflammatory context may predispose CTLs to trigger an exaggerated degranulation activity against stimuli. The above results would indicate that CTL functionality was not impaired in HCV infection.

Concerning peripheral NK cells, a significant decrease of peripheral NK cells was observed in CHC samples of patients, which is in accordance with many authors (Oliviero et al., 2009; Dessouki et al., 2010; Podhorzer et al., 2017). It is proven that NK cells can be classified into two subpopulations with complementary mechanisms, the NK *bright* and the NK *dim*, and an imbalance between them could affect liver damage generation (Yoon et al., 2016). In this study an altered balance between peripheral NK subpopulations, with an increase of NK *bright* and a decrease in NK *dim* was found as described by other authors (Bonorino et al., 2009; Dessouki et al., 2010; Podhorzer et al., 2017). Given different functions of these two subpopulations, we evaluated whether the observed altered proportion resulted in a diminished cytotoxic activity and an augmented IFN-**γ** production capacity or if their functions were conserved. Both total NK cells and NK subpopulations showed conserved IFN-**γ** production capacity and degranulation activity in CHC patients as previously described by Varchetta et al. (2012). Interestingly, those cases with more severe fibrosis presented lower basal degranulation percentages interpreted as fewer cells that spontaneously degranulated, but they showed higher basal levels of CD107a expression intensity perhaps as a compensatory mechanism.

In conclusion, according to our results total portal lymphocytes contributed to the establishment of the inflammatory liver microenvironment, as reflected by liver infiltrates, and participated in the delicate balance of promoting and moderating liver injury in CHC. The presence of liver CTLs, despite inhibiting viral replication, would favor liver damage. Additionally, as a consequence of liver inflammation and cytokine augmentation at the site of infection, the presence of intrahepatic Treg may suggest their regulatory function to ameliorate liver damage. However, after a long inflammatory process, these cells seemed to be not fully efficient in protecting the final worsening of the liver parenchyma. In turn, despite their low number, Th17 lymphocytes tended to promote fibrogenesis. Furthermore, although the manifestations of chronic infection were mainly at the hepatic level, and the peripheral immune response would not have a clear role in generating liver damage, the peripheral alterations would contribute to the pathogenesis of the global disease. Indeed, the T lymphocyte differentiation stage alteration, the plasma cytokine levels, as well as the decrease in NK cells, could condition the establishment of an effective response against other pathogens or even favor the development of extrahepatic manifestations.

The availability of liver biopsy samples from naïve of treatment patients allowed the evaluation of microenvironment features at baseline. Understanding which immune components in the cellular repertoire are important for successful immune responses lays the foundation for future studies evaluating CHC patients during and after DAA treatment in order to understand the new immune scenario when the virus is no longer found. Our results could help to address several open questions remaining for the management of HCV infection in a comprehensive manner.

## Data Availability Statement

The original contributions presented in the study are included in the article/**Supplementary Material**. Further inquiries can be directed to the corresponding author.

## Ethics Statement

The studies involving human participants were reviewed and approved by Ricardo Gutierrez Children Hospitals’ ethics committees. The patients/participants provided their written informed consent to participate in this study.

## Author Contributions

DAR and CGG designed and performed research, analyzed data and wrote the manuscript. PV obtained funding, designed, and performed research, analyzed data, performed statistical analysis and corrected the manuscript. MSC and MIG performed flow cytometric assay and analysis. PCC and BA participated in the drafting of the work and assisted in clinical data interpretation and analysis. ENDM performed histological characterization and immunostaining evaluation. MVP obtained funding, designed research, analyzed data and corrected the manuscript. All authors contributed to the article and approved the submitted version.

## Funding

This work was funded in part by grants from the National Agency for Scientific and Technology Promotion (ANPCyT) (PICT 2014 N°1144, PICT 2014 N°1553, PICT 2017 N°713), MINCYT (Argentina)-CONACYT (Mexico) (ME/13/43) and National Research Council (CONICET) (PIP 2014). CG is a CONICET doctoral fellow. PV, MP, PC, and EM are members of the CONICET Research Career Program. EM is a member of the Research Career of Buenos Aires City Government.

## Conflict of Interest

The authors declare that the research was conducted in the absence of any commercial or financial relationships that could be construed as a potential conflict of interest.

## Publisher’s Note

All claims expressed in this article are solely those of the authors and do not necessarily represent those of their affiliated organizations, or those of the publisher, the editors and the reviewers. Any product that may be evaluated in this article, or claim that may be made by its manufacturer, is not guaranteed or endorsed by the publisher.
