# Supplementary material for: Chronic Hepatitis C Pathogenesis: Immune Response in the Liver Microenvironment and Peripheral Compartment
Source: Front Cell Infect Microbiol. 2021 Aug 3;11:712105. doi: 10.3389/fcimb.2021.712105 (PMC8369367; doi:10.3389/fcimb.2021.712105)
Supplement: Supplementary file 2 [file Presentation_1.pdf]

Supplementary Material

Gating strategies for flow cytometry analysis corresponding to the peripheral lymphocyte populations quantification, T lymphocyte differentiation and functional characterization of CTLs and NK cells.

Gating strategies for flow cytometry analysis.

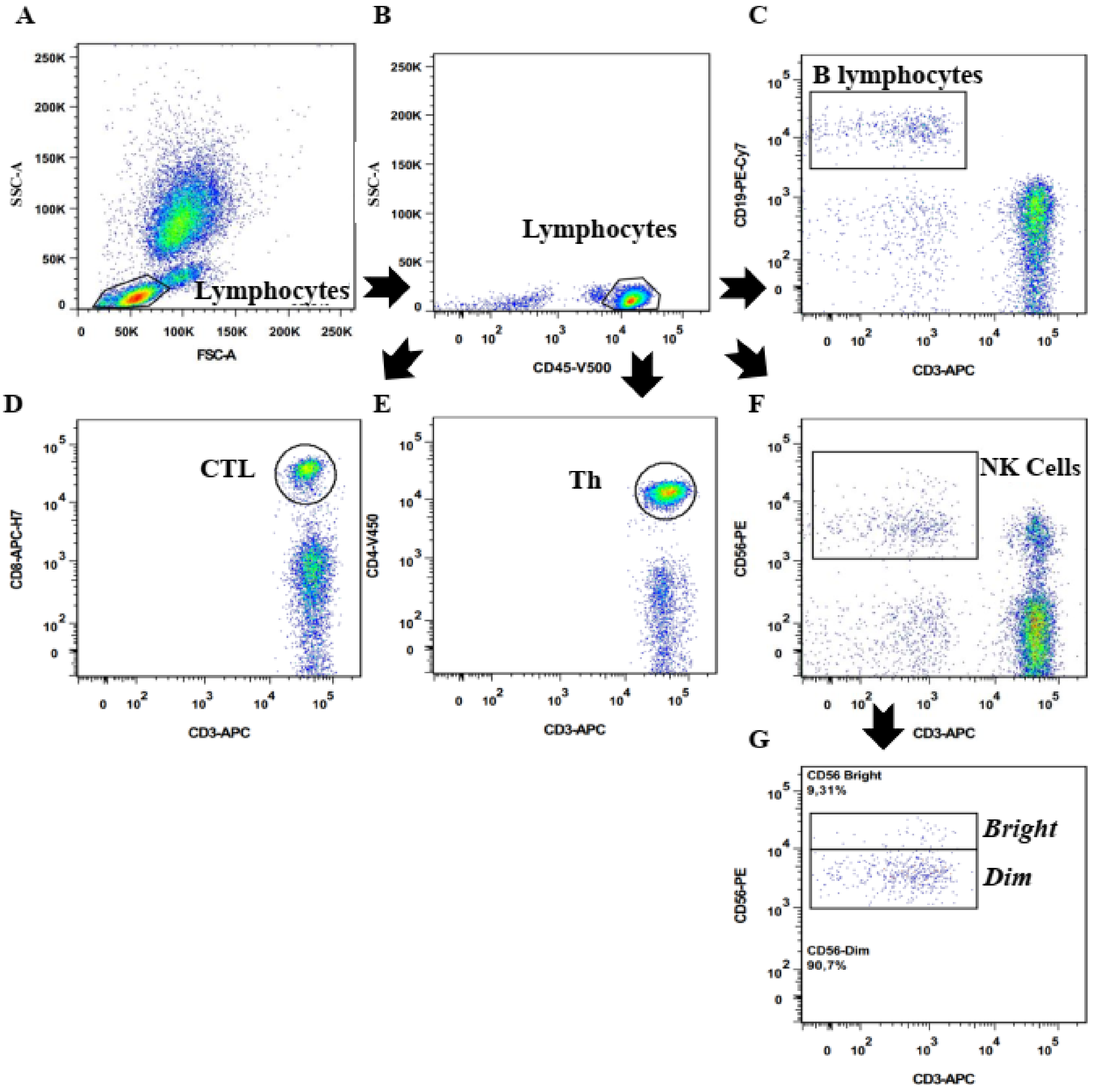

B lymphocytes, Th cells, CTLs and NK cells frequencies on fresh heparinized blood. Lymphocyte selection by SSC-A and FSC-A (A), and CD45 expression (B). Lymphocyte characterization by (C) CD3-CD19+ (B lymphocytes), (D) CD3+CD8+ (CTL) and (E) CD3+CD4+ (Th cells). Finally, NK cells were identified as CD3-CD56+ (F) and discriminated according to CD56 expression in *Dim* and *Bright* (G). 3,000 events were acquired at the CD45+ lymphocyte gate under SSC-A.

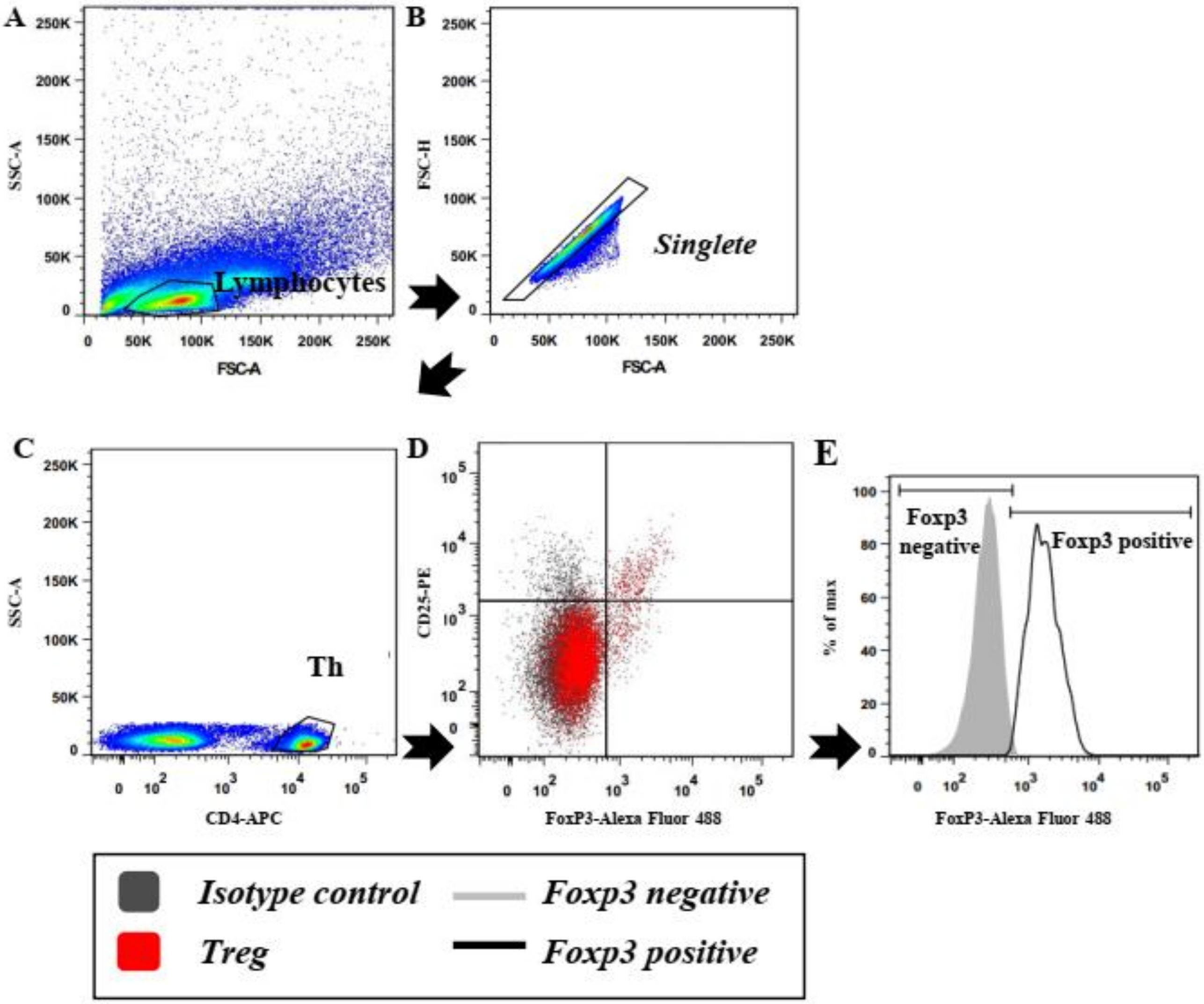

Treg subpopulations on PBMC sample. (A) lymphocyte selection was performed by SSC-A and FSC-A, then the doublets were removed (B). CD4+ cells selection (C) for Treg characterization CD4+CD25hiFoxp3+ cells identification (red) (Isotype control staining is also shown in gray) (D). 3,000 events were acquired at the Th gate. The histogram (E) shows the frequency of events in the Treg tube as a function of the fluorescence intensity of Alexa-Fluor-488 (Foxp3) of the positive Foxp3 population (black) and the negative population (gray). From each peak the geometric mean (MFI) of Foxp3 expression was calculated to express the results as nMFI (positive MFI/negative MFI).

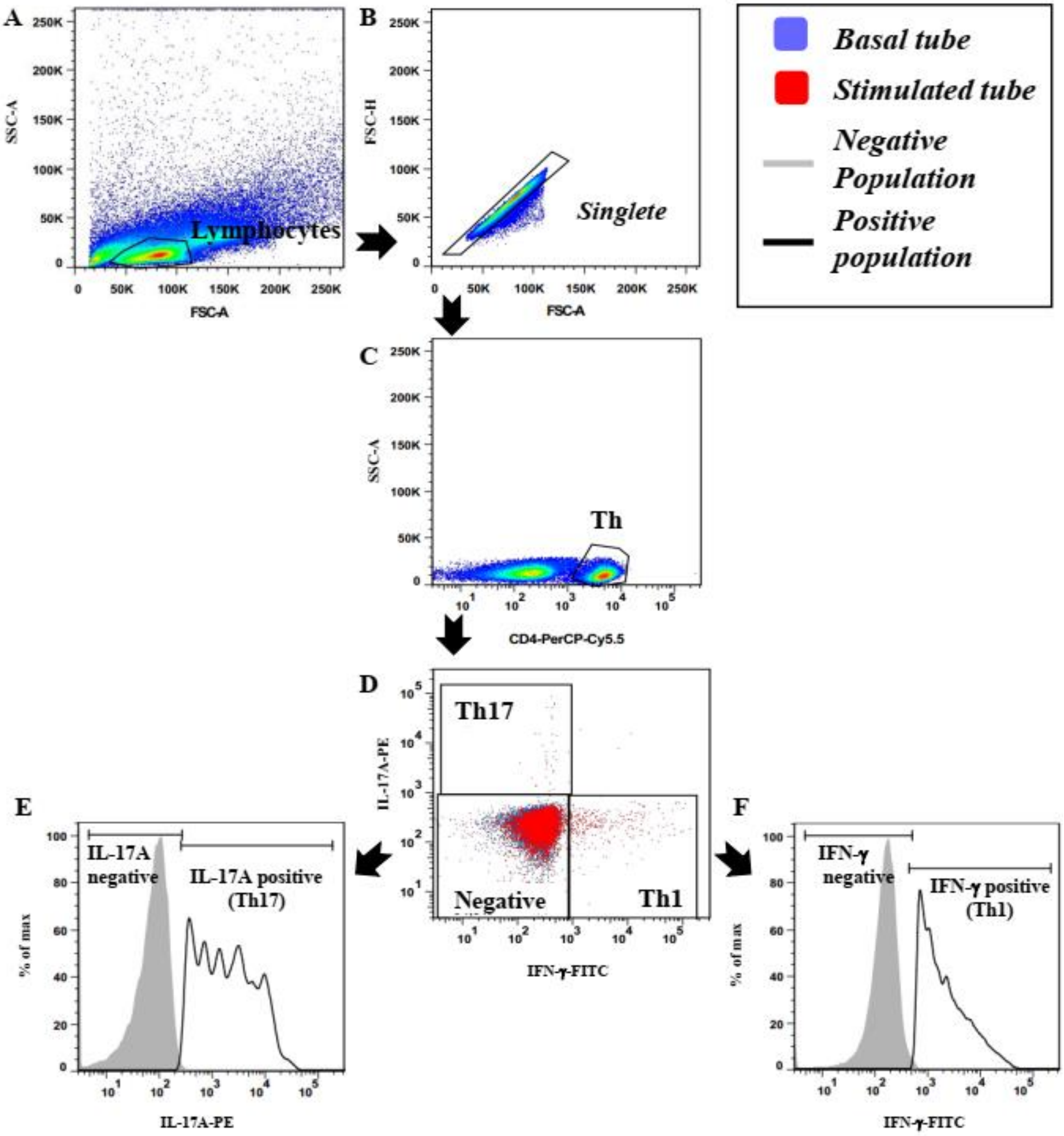

Th1 and Th17 subpopulations on PBMC sample. (A) lymphocyte selection was performed by SSC-A and FSC-A, then the doublets were removed (B). CD4+ cells selection (C) for Th1 and Th17 characterization; (D) CD4+/IFN-γ+ (Th1) cells and CD4+/IL-17A+ (Th17) cells identification after anti-CD3/IL-2 stimulation (red) (unstimulated PBMCs are also shown in blue). 3,000 events were acquired at the Th gate. The histograms (E and F) show the frequency of events as a function of the fluorescence intensity (E: IL–17A; F: IFN–γ) of the positive population (black) and the negative population (gray) in the stimulated tube. From each peak the geometric mean (MFI) of cytokine expression was calculated to express the results as nMFI (positive MFI/negative MFI).

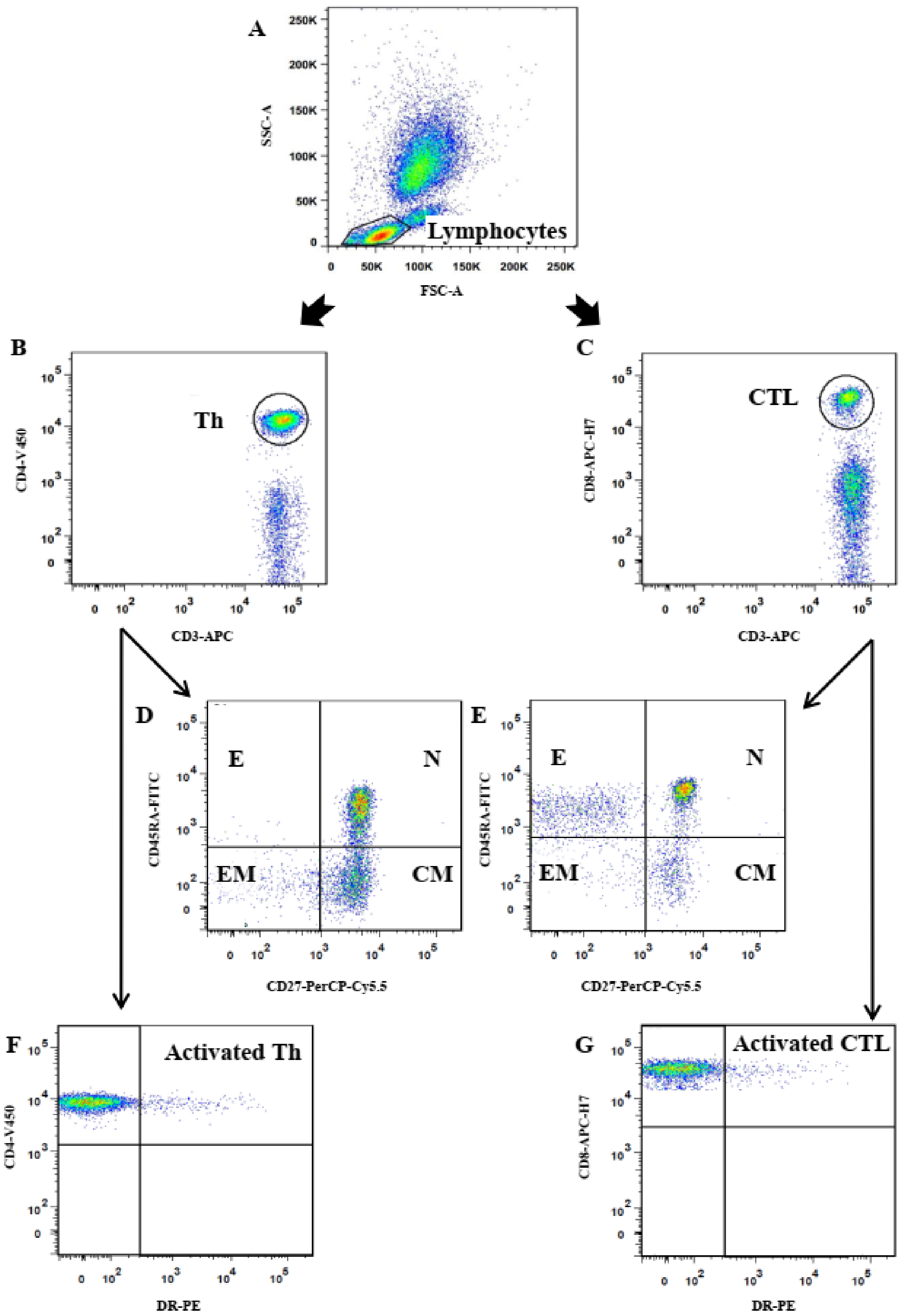

T lymphocyte differentiation frequencies on fresh heparinized blood. Lymphocyte selection by SSC-A and FSC-A (A). Lymphocyte characterization by CD3+CD4+ (Th cells) (B) and CD3+CD8+ (CTL) (C). In both cases, the Naïve lymphocytes (N; CD45RA+CD27+), Central Memory (CM; CD45RA-CD27+), Effector Memory (EM; CD45RA-CD27-) and Effectors (E; CD45RA+CD27-) (D and E) were determined. In addition, the frequency of activated T lymphocytes (HLA-DR+) (F and G) was determined. 3,000 events were acquired at the lymphocyte gate.

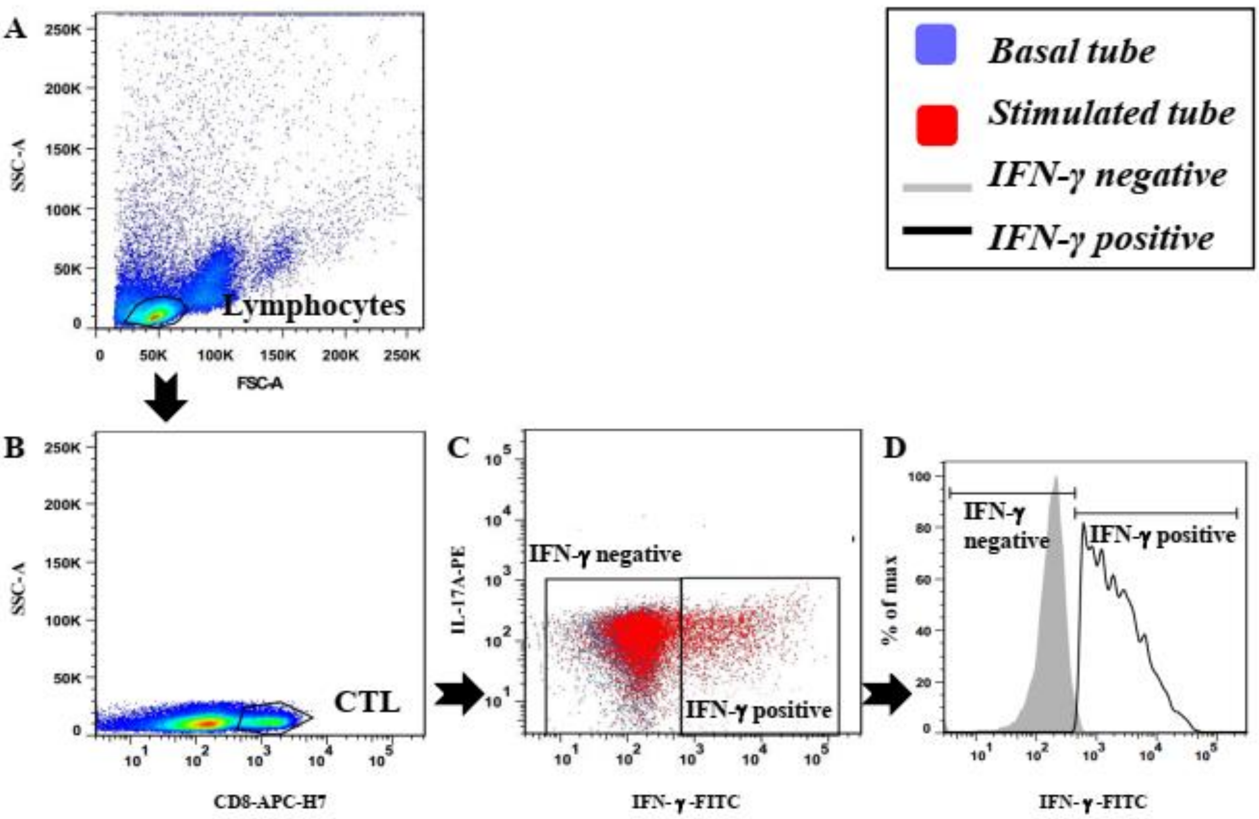

IFN-γ-producing CTL population on PBMC sample. (A) lymphocyte selection by SSC-A and FSC-A, then CD8+ cells selection (B); IFN-γ+ cells identification after anti-CD3/IL-2 stimulation (C) (red) (unstimulated PBMCs are also shown in blue). 3,000 events were acquired at the lymphocyte gate. The histogram (D) shows the frequency of events as a function of the fluorescence intensity (IFN–γ) of the positive population (black) and the negative population (gray) in the stimulated tube. From each peak the geometric mean (MFI) of IFN–γ expression was calculated to express the results as nMFI (positive MFI/negative MFI).

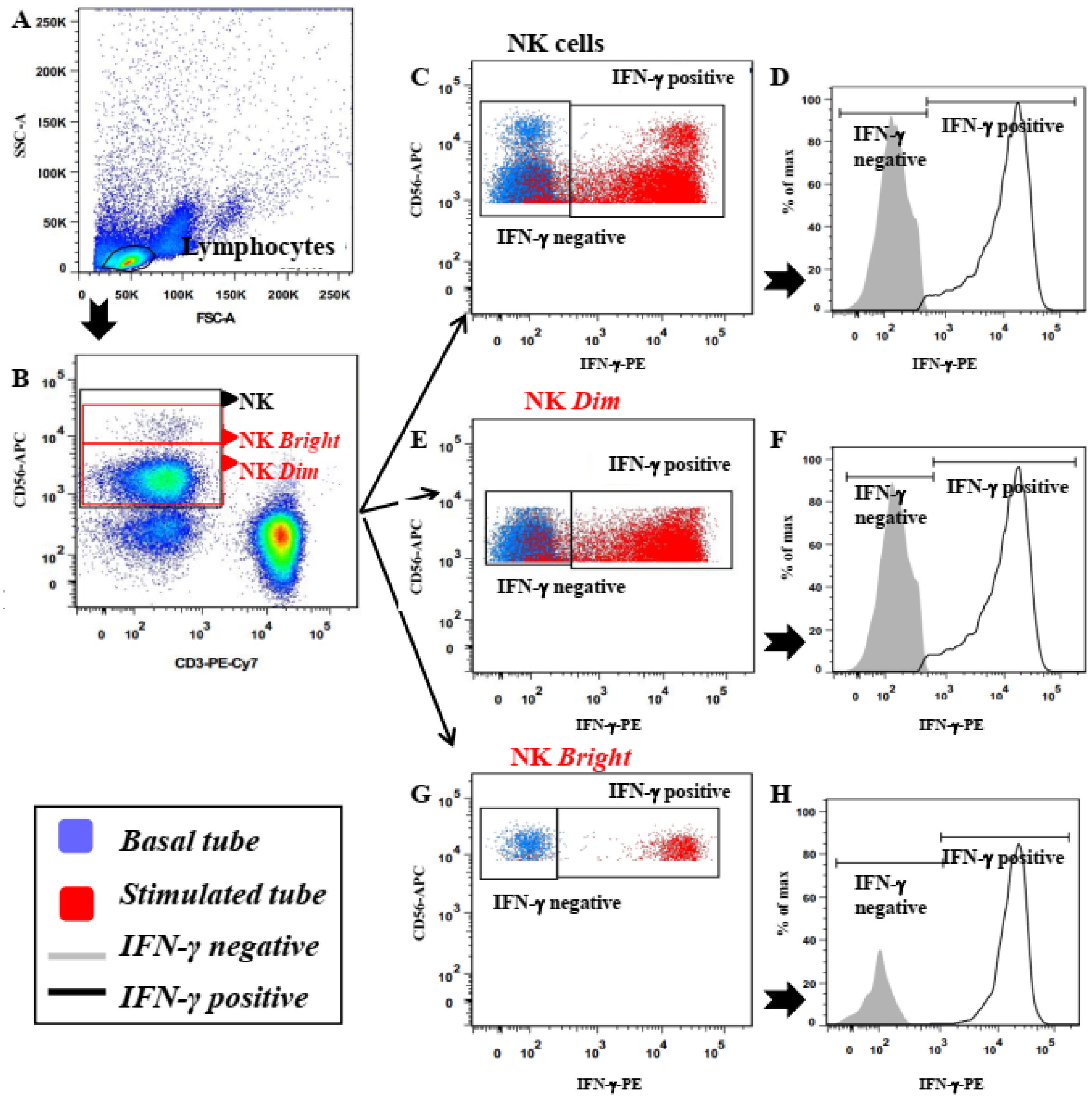

Gating strategies for flow cytometry analysis. IFN-γ-producing NK cell population on PBMC sample. (A) lymphocyte selection was performed by SSC-A and FSC-A, then NK cells were identified as CD3-CD56+ (black box) and their *Dim* and *Bright* subpopulations (red boxes) were discriminated according to CD56 expression (B). IFN-γ+ cells identification after IL–12/IL–15/IL–18 stimulation (red) for total NK cells (C), *Dim* (E) and *Bright* (G) subpopulations were determined (unstimulated PBMCs are also shown in blue). 15,000 events were acquired at the NK CD3-CD56+ gate. The histograms (D, F and H) show the frequency of events as a function of the fluorescence intensity (IFN–γ) of the positive population (black) and the negative population (gray) in the stimulated tube. From each peak the geometric mean (MFI) of IFN–γ expression was calculated to express the results as nMFI (positive MFI/negative MFI).

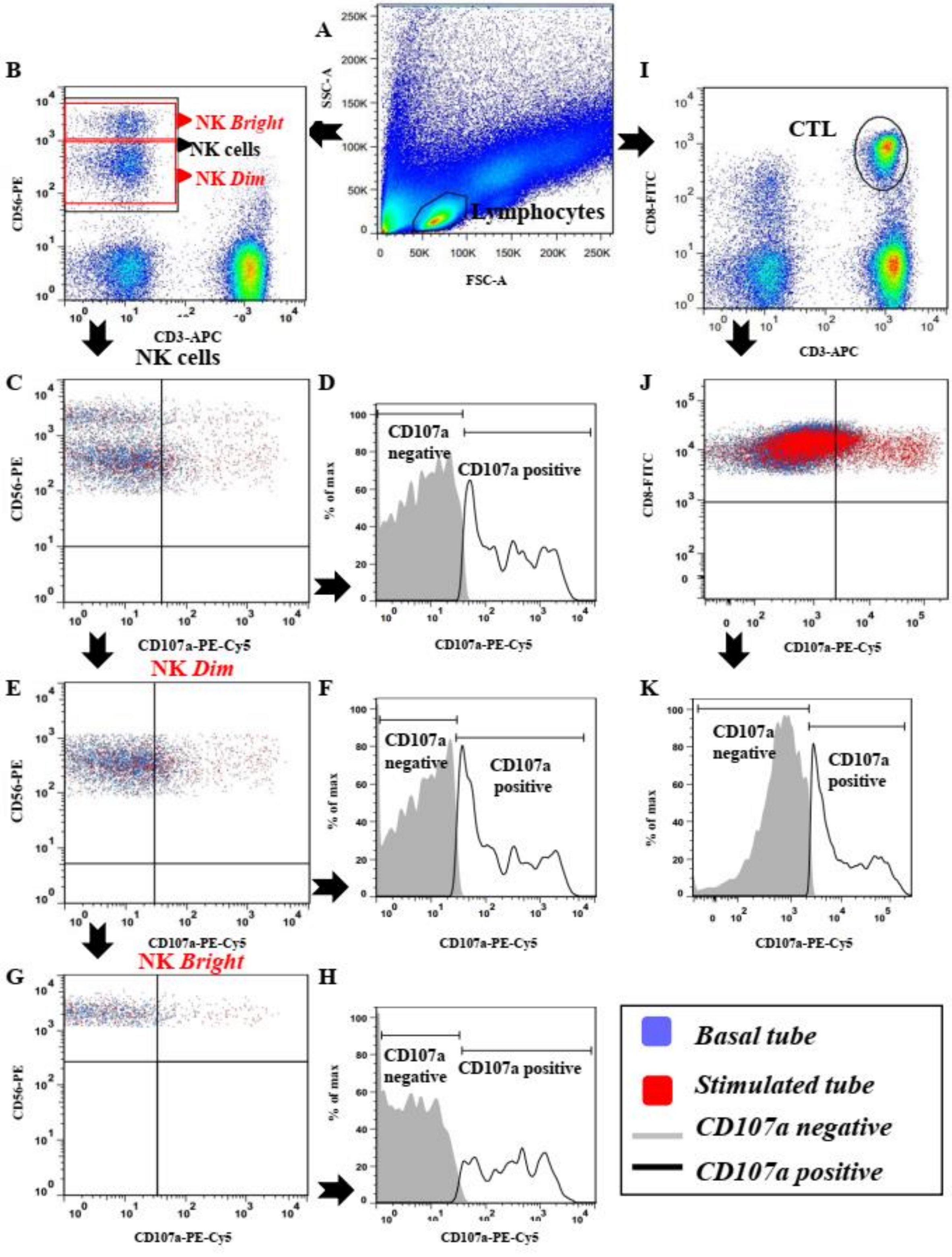

CD107a expression of NK and CTL cells on PBMC sample. (A) lymphocyte selection by SSC-A and FSC-A. Then NK cells were identified as CD3-CD56+ (black box) (B) and were discriminated according to the expression of CD56 its *Dim* and *Bright* subpopulations (red boxes); and CTLs as CD3+CD8+ (I). CD107a expression was determined after the corresponding stimulus (red) in NK cells (C), in *Dim* (E) and *Bright* (G) subpopulations, and in CTL (J) (unstimulated PBMCs are also shown in blue). 5,000 events were acquired at the NK CD3-CD56+ gate. The histograms (D, F, H y K) show the frequency of events as a function of the fluorescence intensity (PE-Cy5 CD107a) of the positive population (black) and the negative population (gray) in the stimulated tube. From each peak the geometric mean (MFI) of IFN–γ expression was calculated to express the results as nMFI (positive MFI/negative MFI).
