# Supplementary material for: Chronic Hepatitis C Pathogenesis: Immune Response in the Liver Microenvironment and Peripheral Compartment
Source: Front Cell Infect Microbiol. 2021 Aug 3;11:712105. doi: 10.3389/fcimb.2021.712105 (PMC8369367; doi:10.3389/fcimb.2021.712105)
Supplement: Supplementary file 3 [file Presentation_2.pdf]

Supplementary Figures and tables

Supplementary Figures 1-5

Supplementary Tables 1 and 2

Supplementary Table 3 shows the data set analyzed during the current study, but as it is an Excel file, is attached as a separate file.

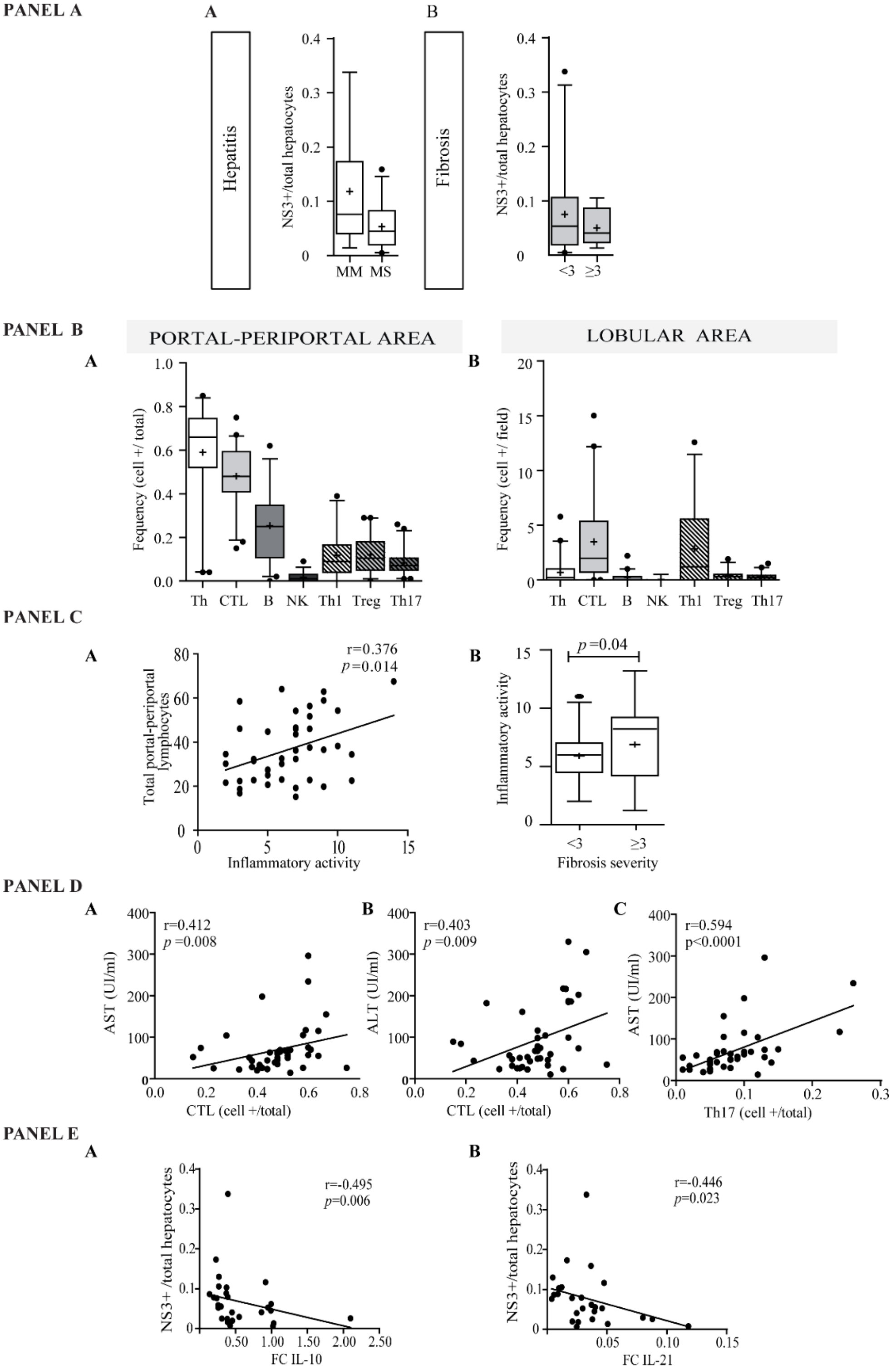

SUPPLEMENTARY FIGURE 1. PANEL A. Infected hepatocytes in liver biopsies of CHC patients. NS3+ hepatocyte related to hepatitis (A) and fibrosis (B). **PANEL B. Frequency and distribution of the immune cells within liver infiltrate.** Portal-periportal (A) and lobular (B) cell population frequencies. PANEL C. Inflammatory activity related to total portal-periportal lymphocyte and fibrosis severity. Correlation between inflammatory activity and total porta-periportal lymphocytes

(A). Inflammatory activity in relation to fibrosis severity (B). **PANEL D. Relationship between portal-periportal CTL and Th17 frequency and biochemical parameters of liver damage.** Correlations between aspartate transaminase (AST) or alanine transaminase (ALT) levels and CTL frequency (A and B) and Th17 frequency (C). **PANEL E. Relationship between liver infection and the expression of IL−10 and IL−21.** Correlation between infected hepatocytes (NS3+) and the hepatic expression levels of IL−10 (A) and IL−21 (B). FC: fold change. When it corresponds, the results are depicted in box plots. Horizontal lines within boxes indicate medians. Horizontal lines outside the boxes represent the 5 and 95 percentiles. Mean is indicated as +. MM: minimal – mild, MS: moderate – severe hepatitis. Advanced fibrosis (F≥3) according to METAVIR. Mann-Whitney U test and Spearman's nonparametric correlation were used to compare data sets.

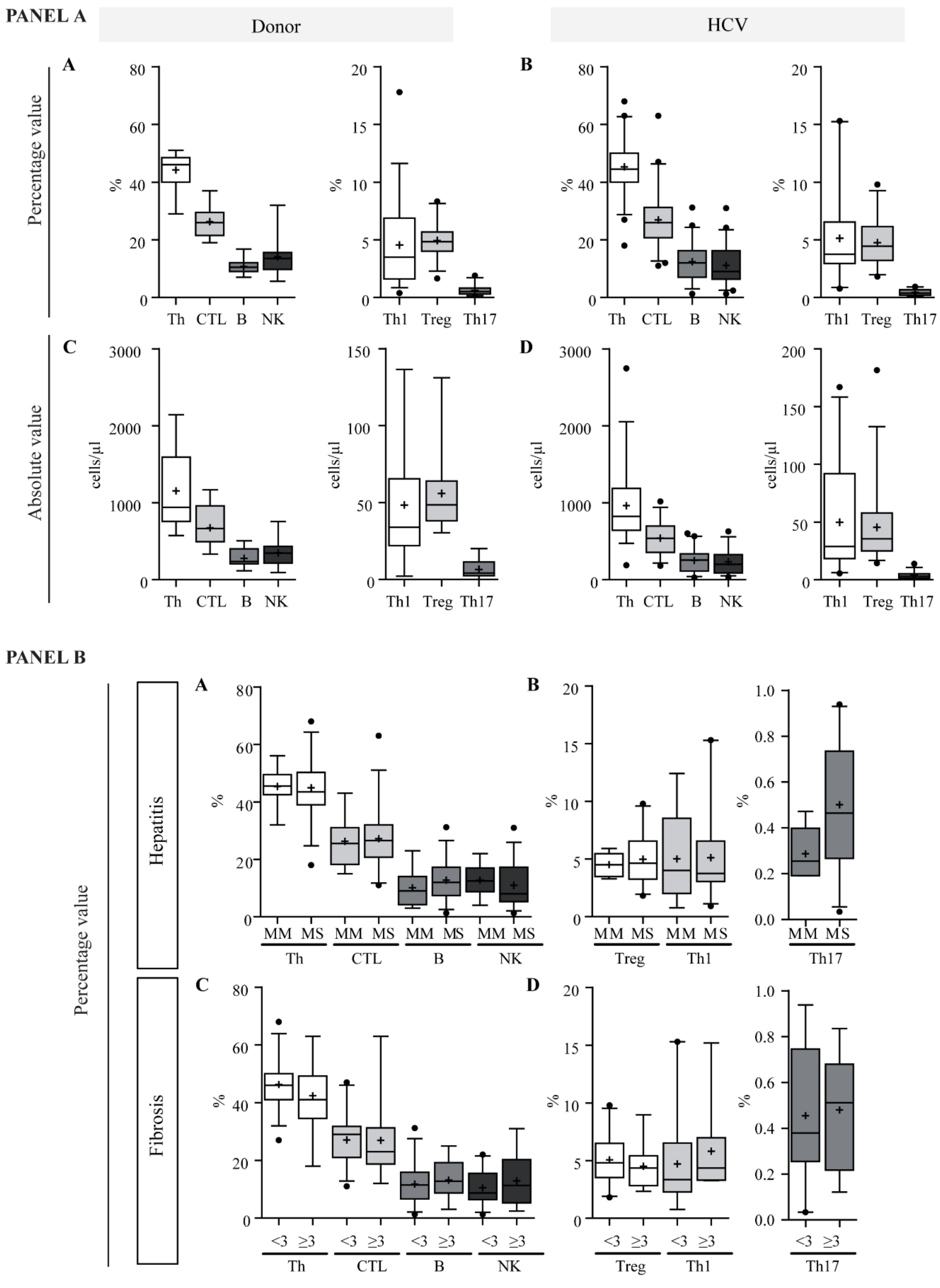

SUPPLEMENTARY FIGURE 2. PANEL A. Peripheral cell populations profile in patients and donors. Percentage (upper) and absolute (lower) values of peripheral cell populations in donors (A, C) and patients (B, D). **PANEL B. Peripheral cell populations related to liver damage.** Percentage

values related to hepatitis (A, B) and fibrosis (C and D) severity. MM: minimal – mild, MS: moderate – severe hepatitis. Advanced fibrosis (F≥3) according to METAVIR. The results are depicted in box plots. Horizontal lines within boxes indicate medians. Horizontal lines outside the boxes represent the 5 and 95 percentiles. Mean is indicated as +. Mann-Whitney U test was applied to compare data sets.

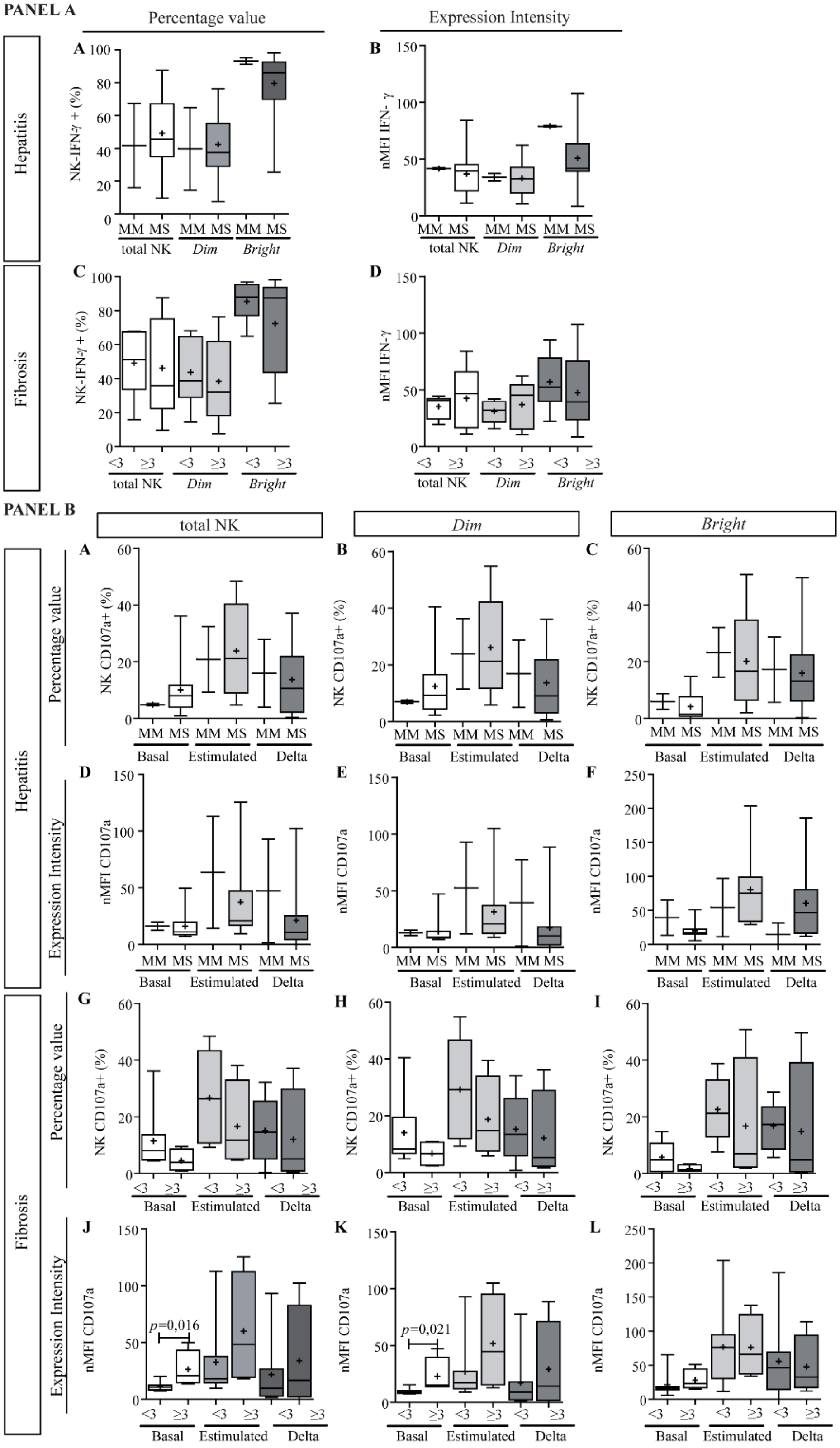

SUPPLEMENTARY FIGURE 3. NK functionality related to liver damage. PANEL A. IFN−γ production capacity of NK cells related to hepatitis (A, B) and fibrosis (C, D) severity. Percentage values (A, C) and intensity of expression (B, D). **PANEL B. Degranulation activity of NK cells**

related to hepatitis (A-F) and fibrosis (G-L). CD107a expression in total NK cells (left), NK Dim (middle) and NK Bright (right). Percentage values (A-C, G-I) and intensity of expression (D-F, J-L). MM: minimal – mild, MS: moderate – severe hepatitis. Advanced fibrosis (F≥3) according to METAVIR. The results are depicted in box plots. Horizontal lines within boxes indicate medians. Horizontal lines outside the boxes represent the 5 and 95 percentiles. Mean is indicated as +. Mann-Whitney U test was applied to compare all data sets. *Note: the analysis of CD107a expression absolute values yielded results similar to the percentage values, but it is not shown to simplify their visualization.*

PANEL A

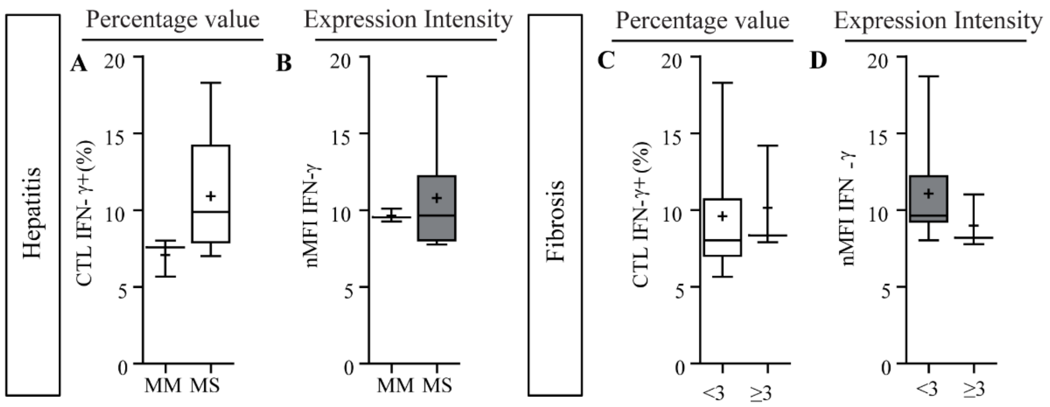

PANEL B

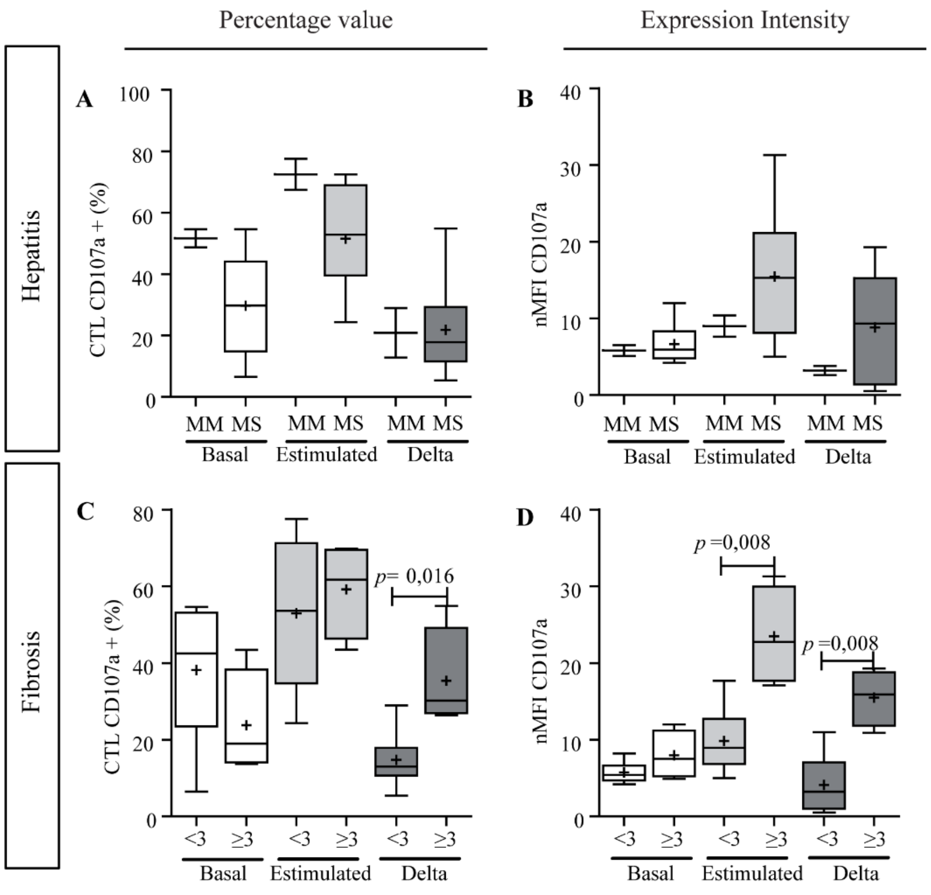

SUPPLEMENTARY FIGURE 4. PANEL A. CTLs functionality related to liver damage. IFN−γ production capacity of CTLs related to hepatitis (A, B) and fibrosis (C, D) severity. Percentage values (A, C) and intensity of expression (B, D). **PANEL B. Degranulation activity of CTLs related to hepatitis (A, B) and fibrosis (C, D).** Percentage values (A, C) and intensity of expression (B, D). MM: minimal – mild, MS: moderate – severe hepatitis. Advanced fibrosis (F≥3) according to METAVIR. The results are depicted in box plots. Horizontal lines within boxes indicate medians. Horizontal lines outside the boxes represent the 5 and 95 percentiles. Mean is indicated as +. Mann-Whitney U test was used to compare all data sets. *Note: the analysis of CD107a expression absolute values displayed similar results to the percentage values, but it is not shown to simplify their visualization.*

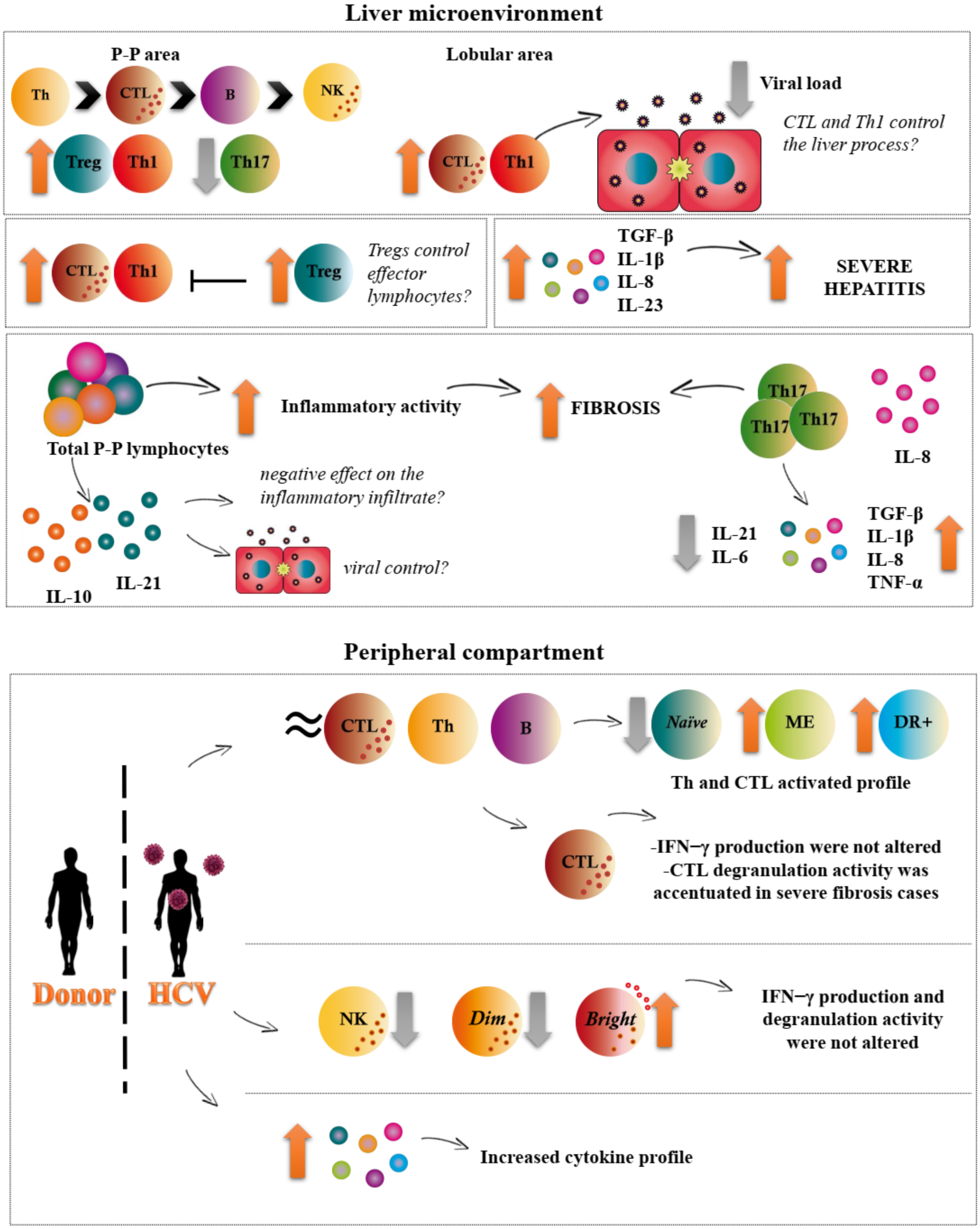

SUPPLEMENTARY FIGURE 5. Schematic representation of the results

SUPPLEMENTARY TABLE 1. Clinical, biochemical, virological and histological patient features.

Patients	Gender	Age (ys)	AST (UI/L)	ALT (UI/L)	Viral genotype	Viral load (UI/ml)	Risk factor for HCV infection	Knodell (HAI)	METAVIR (A-F)
**1**	F	54	64	78	1b	1 150 000	T	6 (3+3)	1-3
**2**	M	57	65	74	1b	1 832 723	OE	10 (6+4)	1-3
**3**	M	49	30	50	1a	48 800	DA	3 (2+1)	1-1
**4**	M	64	33	25	1a	98 797	Ta	7 (5+2)	1-2
**5**	F	57	68	104	1b	1 632 520	D	9 (7+2)	2-2
**6**	F	63	26	34	1b	12 535	T	7 (5+2)	2-2
**7**	M	50	69	59	1a	25 634	D	14 (9+5)	2-3
**8**	F	56	50	32	3a	1 120 040	D	8 (2+6)	1-4
**9**	F	69	198	161	1a	382 702	H	5 (2+3)	1-2
**10***	M	46	173	254	1a	156 123	DA	ND	ND
**11**	F	48	45	98	2	123 452	D	11 (10+1)	2-1
**12**	M	48	75	185	1a	2190 885	DA	7 (5+2)	1-1
**13***	F	51	130	146	3a	698 705	S	ND	ND
**14**	M	35	105	217	1a	1130 289	T	8 (7+1)	2-1
**15**	F	38	50	67	1a	234 563	Se	7 (5+2)	1-1
**16**	F	47	63	42	1a	1 077 776	DA	13 (9+4)	2-3
**17**	M	67	70	186	1b	15 400	D	6 (4+2)	1-2
**18**	M	32	55	202	1b	185 000	D	9 (6+3)	2-2
**19**	F	72	56	45	1b	9 070 000	T	8 (6+2)	2-1
**20**	M	62	117	216	1b	1 550 000	D	14 (10+4)	2-3
**21**	M	54	89	86	1a	4 485 925	Se	11 (5+6)	2-4
**22**	M	47	56	47	1b	ND	Se	8 (5+3)	2-2
**23**	F	51	22	23	1b	270 902	D	4 (3+1)	1-0
**24**	M	45	234	187	1a	ND	D	15 (11+4)	3-3
**25**	F	55	115	73	1b	3 160 000	T	14 (9+5)	3-3
**26**	M	47	57	99	1B	2 800 000	DA	9 (7+2)	2-1

Supplementary Table 1 continued

**Patients**	**Gender**	**Age** (ys)	**AST** (UI/L)	**ALT** (UI/L)	**Viral genotype**	**Viral load** (UI/ml)	**Risk factor for HCV infection**	**Knodell** (HAI)	**METAVIR** (A-F)
**27**	F	55	ND	ND	4	ND	D	5 (5+0)	1-0
**28**	M	62	74	84	1b	4 250 000	D	10 (7+3)	2-2
**29**	F	72	23	22	2c	ND	D	5 (3+2)	1-1
**30**	F	48	43	47	1a	11 200 000	DA	11 (8+3)	2-3
**31**	F	55	51	56	1a	82 400 000	D	13 (9+4)	2-3
**32**	F	58	20	25	1b	2 110 000	D	5 (3+2)	1-1
**33**	M	55	26	28	1b	ND	D	11 (8+3)	2-2
**34**	F	68	69	49	1b	5 540 000	D	14 (10+4)	3-3
**35**	M	53	60	116	1a	30 500	DA	10 (8+2)	2-1
**36**	M	59	28	31	ND	343	T	9 (6+3)	2-2
**37**	F	41	44	52	1b	21 200 000	T	9 (8+1)	2-1
**38***	F	44	42	56	1a	80 711	D	ND	ND
**39**	F	63	39	67	1a	1 170 000	D	12 (7+5)	2-4
**40**	F	68	296	330	2a/c	20 700 000	D	14 (11+3)	3-2
**41**	M	49	35	48	1a	21 900 000	DA	10 (7+3)	2-2
**42**	M	63	26	23	1b	3 850 000	D	7 (3+4)	1-3
**43***	F	60	105	123	1b	1 060 000	D	ND	ND
**44**	M	67	14	10	1b	ND	T	9 (7+2)	2-1
**45**	M	52	155	305	1a	1 560 000	D	10 (7+3)	2-2
**46**	F	37	52	89	1a	3 520	D	5 (4+1)	1-1
**47**	M	51	25	43	1a	67 500 000	D	10 (8+2)	2-1
**48**	M	52	104	182	3a	2 850 000	DA	19 (14+5)	3-4

F: female, M: male. ND: no data. AST: aspartate aminotransferase, ALT: alanine aminotransferase. Normal ALT and AST levels for adult patients were ≤40 and ≤42 IU/L, respectively when testing was done at 37ºC. Risk factor for HCV infection: T: transfusion, OE: occupational exposure, DA: drug abuse, Ta: tattoo, H: hemodialysis, Se: sexual, D: unknown. METAVIR: A: inflammatory activity; F: fibrosis stage. *****Patients who have a non-evaluable liver biopsy and therefore no information is available about liver damage.

SUPPLEMENTARY TABLE 2. qRT-PCR primers description and annealing temperature.

Target	Primer Sequences (5'-3')	Product length (nt)	Annealing Temperature° (°C)
**TNF-α**	F CTGCTGCACTTTGGAGTGAT<br>R AGATGATCTGACTGCCTGGG	93	60
**IL-23**	F AGAAGCTCTGCACACTGGC<br>R CCACACTGGATATGGGGAAC	109	60
**IFN-γ**	F GAGTGTGGAGACCATCAAGGA<br>R GTATTGCTTTGCGTTGGACA	127	58
**IL-1β**	F AACAGATGAAGTGCTCCTTCCA<br>R GGTGGTCGGAGATTCGTAGC	78	60
**IL-6**	F AGTGAGGAACAAGCCAGAGC<br>R GTCAGGGGTGGTTATTGCAT	99	60
**IL-8**	F AAGTTTTTGAAGAGGGCTGAGA<br>R TTGCTTGAAGTTTCACTGGCAT	91	60
**IL-17A**	F AACGATGACTCCTGGGAAGAC<br>R CCTGGATTTCGTGGGATTGTG	99	60
**IL-21**	F CCAAGGTCAAGATCGCCACA<br>R GGCAGAAATTCAGGGACCAAG	99	60
**IL-10**	F GCTGTCATCGATTTCTTCCC<br>R ACAAAGCCATGAGTGAGTTTGA	111	58
**TGF-β**	F CTTCCAGCCGAGGTCCTT<br>R CCCTGGACACCAACTATTGC	92	58
**HPRT**	F ATGGGAGGCCATCACATTGT<br>R ATGTAATCCAGCAGGTCAGCAA	77	60
**β-actin**	F CCACACTGTGCCCATCTACG<br>R CCGTGGTGGTGAAGCTGTAG	131	60

F: forward primer; R: reverse primer. Thermocycler conditions included 95°C for 10 min, followed by 40 cycles at 95°C for 15 sec and 60 sec at the corresponding annealing temperature. In order to verify the specificity of the PCR products, melting curve analysis was performed from 60°C to 95°C with 0.3°C/sec intervals and stepwise fluorescence acquisition. The efficiency of each qRT-PCR reaction ranged between 0.9 and 1.1.
